# The phenology of *Epilobium hirsutum* L.: assessing marker compounds variability of a pharmaceutically important plant remedy

**DOI:** 10.3389/fphar.2025.1602819

**Published:** 2025-07-15

**Authors:** Olha Mykhailenko, Banaz Jalil, Kateryna Uminska, Liudas Ivanauskas, Zigmantas Gudžinskas, Michael Heinrich

**Affiliations:** ^1^ Pharmacognosy and Phytotherapy Group, UCL School of Pharmacy, London, United Kingdom; ^2^ Pharmaceutical Chemistry Department, National University of Pharmacy, Kharkiv, Ukraine; ^3^ Department of Pharmaceutical Biology, Kiel University, Kiel, Germany; ^4^ Zhytomyr Basic Pharmaceutical Professional College, Zhytomyr, Ukraine; ^5^ Department of Analytical and Toxicological Chemistry, Lithuanian University of Health Sciences, Kaunas, Lithuania; ^6^ State Scientific Research Institute Nature Research Centre, Laboratory of Flora and Geobotany, Vilnius, Lithuania; ^7^ Department of Pharmaceutical Sciences and Chinese Medicine Resources, Chinese Medicine Research Center, College of Chinese Medicine, China Medical University, Taichung, Taiwan

**Keywords:** *Epilobium hirsutum*, phenolic compounds, HPTLC, HPLC, marker compounds, phytochemical variability, phenology, industrial crops

## Abstract

**Background:**

*Epilobium hirsutum* L. (Onagraceae), a perennial medicinal plant, has considerable pharmaceutical value due to its phenolic acids, flavonoids, and ellagitannins, including oenothein A and B. The plant is reported to have therapeutic benefits for several conditions, such as prostate gland, bladder, and hormonal disorders. This study aimed to provide evidence-based data on the chemical composition of the species over a 1-year cycle and define the optimal harvesting period, which is essential to ensure consistency and efficacy in plant-derived products.

**Methods:**

The phytochemical variability of *E. hirsutum* across habitats (mesic grassland, wet grassland, and lake shore), plant parts (leaves and stems), and phenological stages (from April to October) was investigated. Using HPLC and HPTLC methods, 11 pharmacologically active marker compounds were quantified in 78 samples collected every 2 weeks during 2023.

**Results:**

Among the hydroxycinnamic acids, chlorogenic acid was dominant in samples from shaded habitats, with maximum accumulation in samples from leaves during the vegetative phase (up to 2.25 mg/g DW). Flavonoids such as isoquercitrin and hyperoside peaked in leaves from the lake shore and wet grassland habitats during flowering. Oenothein B, a major ellagitannin, showed the highest concentrations in wet grassland leaves during flowering (73.97 mg/g DW).

**Conclusion:**

This study is the first to integrate habitat, phenology, and plant part data to characterise seasonal dynamics of key marker compounds in *E. hirsutum* under natural east-part of the United Kingdom conditions. Shaded, moist habitats were found to promote higher biosynthesis of secondary metabolites, whereas open, dry conditions favoured biomass yield. Distinct seasonal patterns in the accumulation of *β*-hydroxycinnamic acids and ellagitannins provide insight into their physiological functions and potential pharmacological significance. By identifying the dynamics of marker compounds and optimal harvesting periods, the study provides a framework for sustainable industrial practices for pharmaceutical raw material production and supports standardisation in herbal production.

## 1 Introduction

Assessing the composition and variability of marker compounds in plant raw materials based on growth phase (phenology) and habitat is crucial to ensure consistent quality and efficacy. This is especially important in the context of the sustainable use of plant-derived products. Several studies highlighted the need to optimise the collection time of medicinal plants to produce material which is of pharmacopeial standard, as indicated by Wang et al. (2023), Heinrich (2015). Furthermore, in the context of conducting studies using herbal extracts, chemical characterisation of the extracts obtained is essential due to possible variability of relevant metabolites and/or pharmacologically active marker compounds based on environmental factors (Heinrich et al., 2022). Significant phytochemical changes in the composition and levels of secondary metabolites and nutrients can occur in plants (He et al., 2023), leading to changes in reported pharmacological activities (Nurzyńska-Wierdak, 2023), safety issues or the lack of desired pharmacological activities (van Wyk and Prinsloo, 2020). Also, determining the optimal growth stage for harvesting ensures that plants are collected when they contain the highest concentration of target compounds, reducing waste and increasing resource efficiency (Mungwari et al., 2025).

The relative quantity of marker compounds can vary depending on environmental factors such as light conditions in the habitat, agrochemical soil composition and nutrient availability. Of course, different parts of the plant, such as leaves, stems, flowers and seeds, have different profiles of marker compounds. Therefore, extensive research and analysis are required to fully understand the dynamics of marker compound accumulation throughout the growth stages of a species. Climate change in Europe has led to significant temperature increases, influencing plant life cycles and the accumulation of bioactive compounds and optimal collection periods of medicinal plants. Europe has seen marked warming in recent decades. This increase in temperature has been associated with shifts in plant phenology, including earlier leaf emergence and flowering (Vogel, 2022). Many European plant species now flower 1–3 weeks earlier than in previous decades (Körner and Hiltbrunner, 2021).

*Epilobium* L. is one of the most species-rich genera in the Onagraceae, with about 180–200 species (Sheidai et al., 2018), and has a long history of use in traditional medicine across Europe, Asia, and North America. In European herbal medicine, the aerial parts of *Epilobium* species, particularly *Epilobium angustifolium* (fireweed) and *Epilobium parviflorum* Schreb. (small-flower willowherb) have been traditionally used for several conditions, such as prostate and urinary tract disorders, benign prostatic hyperplasia, and gastrointestinal problems (Adamczak et al., 2019; Barbarossa et al., 2025). Although *Epilobium* species are not used as frequently in Traditional Chinese Medicine as in Western European countries, the roots and stems of fireweed are used in Chinese Medicine to treat traumatic injuries and relieve inflammation and menstrual disorders (Deng et al., 2019; Li, 2007). *Epilobium* species are widely used in Canada and Alaska to treat inflammation, burns, boils, ulcers, rashes, mouth ulcers and yeast infections (Hassan et al., 2012). In Turkiye, the infusion of the stem and branches of *E. angustifolium* is used traditionally to purify the blood and for prostate diseases (Karakaya et al., 2020).

As part of the evidence-based pharmacy of these local and traditional uses of *Epilobium* species, several pharmacological studies have been conducted to assess the antiproliferative, anti-inflammatory, antioxidant, and other types of activity of extracts from plants of the genus *Epilobium*. The most studied species is *E. angustifolium* L., also known as *Chamaenerion angustifolium* (L.) Scop. or fireweed, is a perennial herb. Its dried, partially or fully fermented leaves, collected before flowering, have traditionally been used to prepare tea-like beverages. An infusion of *E. angustifolium* inhibited the growth of colon cancer cells (Kowalik et al., 2022). *Epilobium hirsutum* has well-differentiated phenological phases during its annual life cycle, including vegetative growth, flowering, seed dispersal and senescence (Mohammadi Bazargani, Falahati-Anbaran, and Rohloff, 2021). These phases are characterised by changes in growth and development patterns and the intensity of accumulation of specific marker compounds. The plant contains a rich profile of secondary metabolites, including flavonoids, phenolic acids, and ellagitannins. These compounds vary across organs, habitats, and phenological stages. To understand the connection between growth stages and compound accumulation, it is essential to consider the plant’s phenological cycle. Therefore, choosing the time and place of collection and the part of the plant to achieve the desired therapeutic effect will help to optimise resource use. For *E. hirsutum*, ongoing changes in climate mean that the optimal timing of accumulation of bioactive compounds, in particular flavonoids and ellagitannins, may shift, requiring adaptive harvesting strategiesThe chemical composition of *E. hirsutum* depends on the stage of development, the geographical origin, and the conditions of primary processing of the plant materials (Mohammadi Bazargani, Falahati-Anbaran, and Rohloff, 2021). Plants of the genus also show variations in the quantitative content of oenothein B depending on the species (Ducrey et al., 1997; Vlase et al., 2022). At the same time, the content of oenothein B in *E. hirsutum* is almost three times higher than in other species (Vlase et al., 2022), which makes this species more suitable for pharmaceutical development rather than for use as a tea infusion. Based on its importance as a herbal medicine and well-documented chemical composition, it offers an interesting case study to assess the chemical composition of the phenological cycle of the species.

Flavonoids, including quercetin, kaempferol, and myricetin derivatives, are key secondary metabolites in *Epilobium* species and are also relevant as marker compounds. The length of the growing season, light intensity, and soil nutrients influence their accumulation. Samples from several regions of Europe, such as Poland and Lithuania, have been reported to accumulate higher levels of myricetin, possibly due to lower temperatures and different soil pH conditions (Lasinskas et al., 2024). Altitude also influences flavonoid biosynthesis in the aerial parts of *E. angustifolium*, and quercetin-3-O-glucuronide is a potential marker for this trend (Monschein et al., 2015).

In addition, these species also contain triterpenoids, amino acids, organic acids and other metabolites that potentiate the action of tannins (Uminska et al., 2023; Bazylko, Kiss, and Kowalski, 2007). Oenothein B is the predominant polyphenol in this genus (14%–23%), while the flavonoid content was less than 2% (Kiss, Kowalski, and Melzig, 2004).

Ethnopharmacologically application for urinary and prostate disorders, *Epilobium* species have shown anti-inflammatory, antiproliferative, and antioxidant effects. Oenothein B, a dominant tannin, has been linked to various biological activities including immunomodulation and cytotoxicity toward cancer cells. The potential therapeutic activity of *Epilobium* species has been linked to the high content of ellagitannins (oenotheins A and B) and epigallocatechin gallate (Abid, Kanwal, and Qaiser, 2014). Oenothein B has several potential reported activities, including antioxidant, immunomodulatory, cytotoxicity of tumour cells, and inhibition or induction of enzyme activity (Nowak et al., 2021; Karakaya et al., 2020). Much of the research on oenothein B has focused on its effects on abnormal prostate cells, it inhibits cell proliferation, prostate-specific antigen (PSA) secretion, and arginase activity *in vitro* (Stolarczyk et al., 2013).

This study aimed to analyse the content of marker and other biologically active metabolites in *E*. *hirsutum* at different developmental stages and growing under different habitat conditions to improve our understanding of phytochemical variability of plant in relation to habitat and phenological stage, which may inform future applications in raw material quality assessment and to facilitate resource-efficient harvesting methods. The study’s originality lies in its fine-scale, environment-dependent, and phenology-linked mapping of metabolite accumulation, using complementary chromatographic techniques (High Performance Thin Layer Chromatography (HPTLC) and High Performance Liquid Chromatography (HPLC)) to generate detailed profiles of plant’s compounds. This approach provides novel insights into how environmental and developmental factors shape secondary metabolite composition in *E. hirsutum*, which may inform future pharmacological applications in raw material quality assessment and contribute to the development of adaptive strategies for sustainable use of plant resources and resource-efficient harvesting. This work supports the UN Sustainable Development Goals (SDGs), particularly Goal 12 and Goal 15. In addition, a crucial step towards achieving sustainability in the collection of plant raw materials and the development of substances is Goal 4, which aims to provide adequate knowledge through planned research and to create the opportunity to achieve a high-quality final product while preserving local and global biodiversity (SDG, 2024; Xu and Peng, 2024).

This study aims to address the following key questions regarding the ethnopharmacology and application of *E. hirsutum*: (a) How does habitat variation influence the content of bioactive metabolites? (b) Which plant parts accumulate the highest (or optimal) amount of pharmacologically relevant marker compounds? (c) How does harvest time affect the bioactive metabolite profile and potential therapeutic efficacy? (d) How do these factors interact with the quality, consistency, and pharmacological potential of the raw material?

Taken together, these questions guide research that aims to optimise the collection and use of *E. hirsutum* as a medicinal plant by assessing its phytochemical variation under natural conditions.

## 2 Materials and methods

### 2.1 Study species

*Epilobium hirsutum* L. (Onagraceae) is a perennial herb, usually 1.8–2 m tall, occasionally up to 2.5 m. The stem is strong and densely covered with soft, spreading hairs. Leaves sessile, clasping stem, lanceolate-elliptic to narrowly obovate or elliptic, hairy on both sides. Flowers large, 10–20 mm in diameter, with four pink or purple-pink petals. Stigmas white, with four lobes. Fruit a capsule, 3–9 cm long, usually hairy. Seeds dark brown, 0.8–1.2 mm long. This species is native to Europe, Africa and much of Asia, except most of Siberia. Naturalised in North America. Usually grows in a wide range of open, wet and damp, occasionally mesic habitats up to 2,500 m above sea level.

### 2.2 Study sites

Three large stands of *E. hirsutum*, covering at least 50 m^2^ and occurring in habitats of varying water availability, were selected for plant sampling in this study. All stands of the study species were selected in the vicinity of Wallington, London, United Kingdom, to eliminate the effect of meteorological conditions that may occur at distant sites (Table 1). The average temperature (°C), sum of precipitation (mm) and sunshine duration (s) of the meteorological conditions during the whole study period (from the beginning of April to the end of October 2023) that could have influenced the accumulation of metabolites in *E. hirsutum* were obtained from Sutton, Weather Station from Open-Meteo.com Weather API (Zippenfenig, 2023). Data are given in Supplementary Table S1.

**TABLE 1:** Characteristics of *Epilobium hirsutum* sampling sites in Wallington, United Kingdom.

Site	Coordinates	Habitat	Plant cover (%)		
			Trees	Shrubs	Herbs
Grange Gardens	51.371835°N, −0.150352°W	Mesic grassland	10	10	70
Green Hackbridge	51.377789°N, −0.160898°W	Wet grassland	20	0	70
Manor Garden	51.370086°N, −0.152038°W	Lake shore	0	10	60

The chosen approach of collecting raw materials from natural habitats allowed for indirect control of plants’ growth conditions through the careful selection of sites and systematic recording of environmental and botanical data.

It also ensured the traceability of plant materials (which is not always possible from commercial suppliers) and prevented potential contamination or adulteration. It ensured that the collected plant material accurately reflected natural vegetative and phytochemical changes.

At the Grange Gardens site, the *E. hirsutum* stand occupied mesic fringe grassland surrounded by sparse *Quercus robur* woodland. As a result, the grassland was partially shaded by trees in the morning and evening. The soil at this site was moderately moist, with no standing water on the surface at any time of the year. At the Green Hackbridge site, the study species grew in a wet grassland habitat close to the river. They are surrounded by a stand of *Alnus glutinosa*, which provides light shade to *E. hirsutum* in the morning and evening. The soil at this site was waterlogged in spring, damp after heavy rainfall and wet during the dry months. At the Manor Garden site, the stand of *E. hirsutum* occupied the completely open shore of a eutrophic artificial lake. The roots of the sampled plants were in the waterlogged or damp soil of the lake shore throughout the growing season. Herb cover was similar at all study sites, ranging from 60% to 70% of the surface (Table 1), and the study species represented approximately 30% of all herbs at all sites.

### 2.3 Sampling of *Epilobium hirsutum*

To assess the dynamics and variability of the chemical profile of *E. hirsutum*, plants were sampled at regularly 2-week intervals from the beginning of the growing season at the end of April (28 April 2023) until the onset of wilt at the beginning of October (3 October 2023). Five well-defined phenological phases of *E. hirsutum* were defined: intensive growth, flower formation, flowering, seed dispersal and wilting. The flowering phase was further subdivided into three subphases: beginning of flowering, intensive flowering and end of flowering. Each phenological phase was defined by observable morphological traits (e.g., shoot height, leaf size, presence of flower, seed set) and occurred over a consistent period of time across sites, with only ±3–5 days variation depending on microhabitat conditions (Figure 2). All samples were collected on the same day. For each phenophase, 3–4 individual plants were sampled at each site, ensuring a minimum of three biological replicates per group. All sampled individuals at a given site were synchronized in phenophase to reduce developmental variability, and only individuals at the same stage were included in each sampling. A total of 78 samples of leaf and stem were collected at 13 sampling events, representing all three habitats and all defined phenophases with sufficient biological replication (minimum of three replicates per phenophase per site in almost all cases).

**FIGURE 1:**
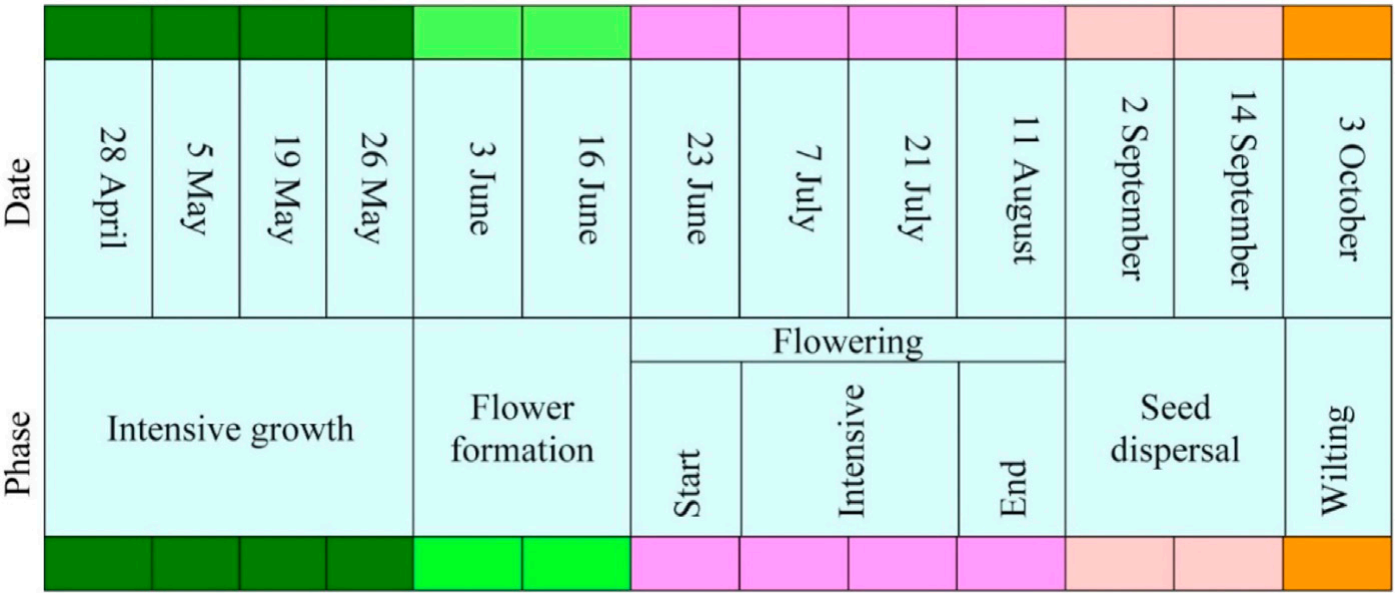
Phenological phases of *Epilobium hirsutum* and dates of sample collection. Different colours distinguish different phenological phases, and this colour chart is used in the graphs to distinguish phenological phases.

**FIGURE 2:**
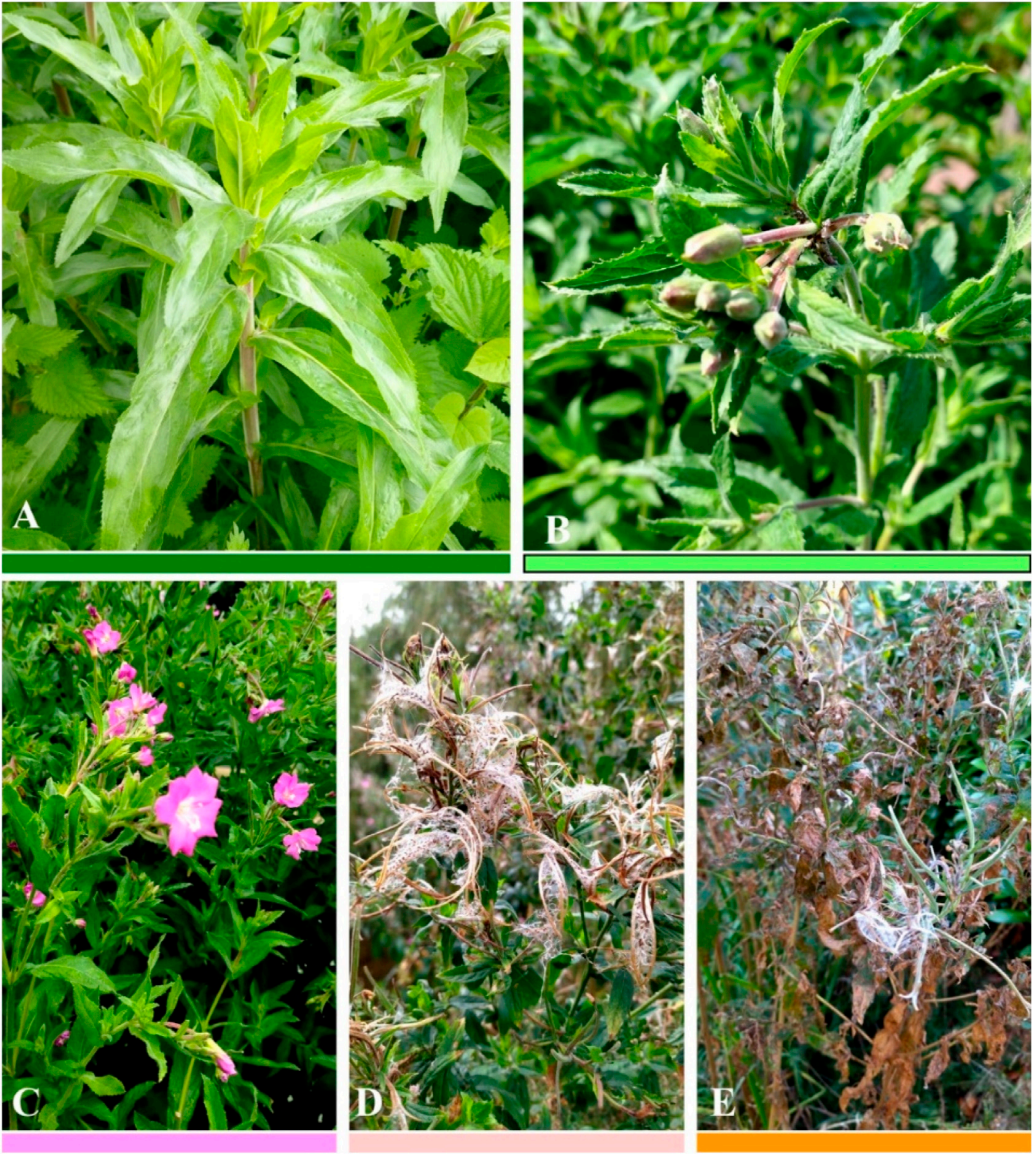
Phenological phases of Epilobium hirsutum at sampling sites in Wallington, United Kingdom. Phenological phases: intensive growth **(A)**, flower formation **(B)**, flowering **(C)**, seed dispersal **(D)**, wilting **(E)**. The colour chart used to indicate phenological phases is the same as in Figure 1. Photos by O. Mykhailenko, 2023.

The plants grew rapidly during the intensive growth phase, which lasted from the end of April to the end of May. At the end of April, the shoots were 10–15 cm high, and a month later, they had grown to 80–100 cm but had not yet started to develop flowers (Figure 1). The upper part of the shoot began to branch. The first flowers began to form in early June, and this phase continued until mid-June (Figure 2). In the second half of June, the plants started to flower, and 2 weeks later, they reached a phase of intensive flowering that lasted until the second half of July. By early August, the flowering of *E. hirsutum* plants had almost ceased, with only single flowers remaining on the main and secondary branches. By early September, all flowers on the main branches had produced fruits, and some of the fruits were already ripe and starting to release seeds (Figures 1, 2). By early October, most of the seeds had been released, and the leaves on the upper part of the plant had begun to turn yellow. A total of 78 *Epilobium hirsutism* samples were collected during the 2023 growing season and used for analysis. Plants were identified by Dr O. Mykhailenko, and voucher specimens (OM1–OM78) were deposited in the herbarium of the School of Pharmacy, University College London.

Plants in wild populations were randomly sampled on dry, windless days. The upper part (approximately two-thirds of the plant) was cut off and labelled, indicating on the label the stage of development, the location and the sampling date. In the laboratory, leaves with flowers were separated from the stems and the collected raw material was dried at an ambient temperature of 20°C–24°C in a well-ventilated room. Thus, each analytical group, representing habitat type and developmental stage, consisted of two samples: leaves (including flowers) and stems. Plant samples were crushed immediately before chemical analysis.

### 2.4 Chemicals and reagents

Gallic acid, ellagic acid, chlorogenic acid, caffeic acid, oenothein B, hyperoside, isoquercitrin (syn. isoquercetin), avicularin, guaijaverin, myricetin were obtained from Inc. ChromaDex (Santa Ana, United States) and Sigma-Aldrich (Saint Louis, United States). All solvents (acetonitrile, methanol, glacial acetic acid) were of HPLC grade and were purchased from Merck KGaA, Fisher Scientific Ltd. And VWR International LLC, except of deuterated methanol, which was purchased from Cambridge Isotope Laboratories Inc. Analytical- and chromatographic-grade chemicals and solvents were used for this study: acetonitrile, methanol, glacial acetic acid from Sigma-Aldrich (GmbH, Karlsruhe, Germany). The water was purified using the ULTRAPURE water system (Millipore, Germany). All other chemicals were of analytical grade.

### 2.5 Preparation of samples and reference standards

For the HPTLC analysis, air-dried powdered plant material (0.50 g) of each sampling date sample was extracted with 50% (v/v) methanol (1:10) in an ultrasound-assisted extraction for 20 min at 40 °C. The sample preparation for HPLC analysis was the following: a precise weight (0.20 g) of each sample powder was extracted with 50% (v/v) methanol (1:50) in an ultrasonic bath (WiseClean) at 45°C ± 2 °C for 20 min. Extraction yields ranged from 7.4% to 6.1% for leaves and from 5.3% to 3.4% for stems extracts. All extracts for both analyses were filtered using a Millex R Syringe filter unit 0.45 mm. The references (avicularin, guajaverin, hyperoside, oenothein B, isoquercitrin, gallic acid, ellagic acid, chlorogenic acid, myricetin) were dissolved in methanol, with a concentration of 1 mg/μL, then sonicated for 10 min. Both the reference standards and the test samples were stored at 4°C.

### 2.6 HPTLC chromatographic analysis

The samples and reference standards were injected individually using a CAMAG Linomat 5 semi-automated sampler (100 μl syringe). The injection volumes of 3 μL reference standards and 7 μL sample solutions were applied. HPTLC glass plates Silica gel 60 F_254_ used for the stationary phase were purchased from Merck KGaA (Darmstadt, Germany). Samples and reference standards were applied to the plates as bands 8 mm wide. The space between bands was 2.0 mm, and the application rate was 90 nL·s^−1^.

Plates were developed in the ADC 2 with chamber saturation (with filter paper) for 20 min and after activation at 33% relative humidity for 10 min using a saturated solution of magnesium chloride, development with ethyl acetate–formic acid–water 68:8:8 (*v/v*) to the migration distance of 70 mm (from the lower edge), followed by drying for 5 min. The temperature and relative humidity were controlled to 21°C–24°C and 33%, respectively. The development distance was 70.0 mm from the lower edge.

A total of 78 methanolic 50% (v/v) extracts (39 leaf and 39 stem samples) were applied across 8 HPTLC plates, with each plate accommodating up to 15 lanes. Each lane represented a different extract corresponding to a specific plant part (leaf or stem), habitat (mesic grassland, wet grassland, lake shore), and phenological stage (from intensive growth to wilting). Reference standards were applied in duplicate to each plate for comparison.

The developed plates were derivatised by dipping in Natural Product A reagent (2 g of 2-aminoetheyl diphenylborinate in 200 mL of methanol) followed by PEG400 (macrogol 400; 10 g polyethylene glycol 400 in 200 mL dichloromethane) using a CAMAG chromatogram immersion device and heated at 100°C on a plate heater for 5 min. The plates were visualised using CAMAG Visualizer under white light, UV 254 nm and UV 366 nm, and the data was analysed using CAMAG visionCATS 3.1 software.

### 2.7 HPLC chromatographic analysis

Chromatographic separation of polyphenols was performed using the Waters e2695 Alliance HPLC system coupled with a 2998 PDA detector (Waters, Milford, MA, United States). Phenolic compounds were separated on an ACE Super C_18_ (250 mm × 4.6 mm, 3 µm) column (ACT, Aberdeen, United Kingdom) with a full run time of 81 min. Column temperature was 25 °C. The gradient elution mode consisting of 0.1% (*v*/*v*) trifluoroacetic acid in pure water (A) and acetonitrile (B) was as follows: 0 min, 5% B; 8–30 min, 20% B; 30–48 min, 40% B; 48–58 min, 50% B; 58–65 min, 50% B; 65–66 min, 95% B; 66–70 min, 95% B; 70–81 min, 5% B. The flow rate was 1 mL/min, and the injection volume was 10 µL. The samples were subjected to two different analyses. The quantity of oenothein A was obtained by recalculation through oenothein B. The quantity of myricetin 3-*β*-D-glucopyranoside (syn. isomyricitrin) was obtained by recalculation through myricetin. Further in the text, the trivial name of the compound–isomyricitrin–will be used in order to maintain uniformity in naming all identified compounds. The analytical HPLC-PDA method for polyphenols was validated according to the ICH Q2 (R1) guidelines and described at (Ivanauskas et al., 2024). The calibration curve, limits of detection (LOD), limits of quantification (LOQ), and the linear range for each analyte are provided in Table 2; precision, repeatability, and recovery for each compound presented in Table 3; and specificity of the compounds is presented in Table 4 and was verified by comparing the retention times and UV-Vis spectra of the compounds in 78 extracts in comparison with the reference standard.

**TABLE 2:** Calibration curves, linearity range, LOD/LOQ, repeatability and precision data of phenolic reference compounds.

Metabolite	RT, min	Coefficient of determination *R* ^2^ ^a^	Calibration curve^a^	Linearity range (µg/mL)	LOD (µg/mL)^b^	LOQ (µg/mL)^c^	Repeatability RT/area (%)	Precision RT/area (%)
Gallic acid	6.10	0.99996	y = 3.39 × 10^4^x + 4.28 × 10^3^	0.68–174.00	0.15	0.66	0.2/0.7	0.3/0.8
Oenothein B	10.66	0.99764	y = 1.81 × 10^4^x−9.31 × 10^3^	0.78–100.00	0.21	0.42	0.1/0.8	0.3/1.2
Chlorogenic acid	11.95	0.99980	y = 3.10 × 10^4^x−7.62 × 10^3^	0.41–208.20	0.05	0.15	0.2/0.6	0.4/0.8
Ellagic acid	22.48	0.99999	y = 1.00 × 105x−6.18 × 10^3^	0.19–23.70	0.07	0.13	0.1/0.3	0.2/0.5
Hyperoside	23.62	0.99985	y = 2.10 × 10^4^x−5.17 × 10^3^	1.51–193.80	0.13	0.38	0.2/0.6	0.3/0.8
Isoquercitrin	24.50	0.99978	y = 2.18 × 10^4^x−7.31 × 10^3^	1.46–187.20	0.28	0.77	0.2/0.7	0.4/0.9
Guaijaverin	28.31	0.99838	y = 1.21 × 10^4^x + 5.33 × 10^3^	1.56–100.00	0.26	0.78	0.1/1.0	0.3/1.2
Myricetin	35.67	0.99991	y = 3.44 × 10^4^x−1.17 × 10^4^	2.13–133.20	0.45	0.45	0.3/0.7	0.4/1.3
Avicularin	30.49	0.99998	y = 2.33 × 104x−5.88 × 10^3^	1.69–430.64	0.23	0.23	0.1/0.6	0.2/1.1

^a^ Concentration of metabolites (mg/mL); y, peak area.

^b^ LOD, limit of detection (S/N = 3).

^c^ LOQ, limit of quantification (S/N = 10).

**TABLE 3:** Precision and stability of phenolic reference compounds.

Compound	Concentration (µg/mL)	Precision				Repeatability	
		Intra-day (*n* = 3)		Inter-day (*n* = 3)		Recovery (%)	RSD (%)
		RSD (%)	Accuracy (%)	RSD (%)	Accuracy (%)		
Gallic acid	7.65	0.57	99.81	0.75	101.37	101.07	0.65
	30.35	0.78	99.56	0.24	102.14	99.69	0.56
	61.20	1.02	101.53	0.38	101.32	100.09	0.94
Oenothein B	3.12	0.72	99.52	0.43	100.12	99.89	0.65
	25.00	1.13	100.23	0.96	99.85	99.97	0.95
	100.00	0.85	99.67	0.98	100.11	97.73	0.68
Chlorogenic acid	5.75	1.31	101.12	0.38	98.40	100.69	0.86
	23.00	0.42	99.08	0.73	99.43	99.58	1.05
	46.00	0.96	100.27	0.48	98.24	101.91	0.97
Ellagic acid	1.48	0.88	100.01	0.48	99.68	99.35	0.63
	5.92	1.01	99.35	0.93	99.87	99.81	0.87
	23.70	0.95	99.76	0.95	100.73	98.98	0.53
Hyperoside	3.433	0.99	101.06	0.86	98.95	100.78	0.90
	13.715	0.50	101.04	0.70	99.07	100.72	0.71
	26.915	0.42	99.53	0.80	100.97	99.77	0.43
Isoquercitrin	5.57	0.86	100.26	0.41	100.23	100.13	0.69
	22.28	1.12	101.27	0.98	99.24	99.64	0.90
	44.56	0.80	99.58	0.91	100.92	97.79	0.49
Guaijaverin	3.12	0.85	100.01	0.47	100.81	100.01	0.63
	12.50	0.92	101.03	0.96	99.97	99.45	0.89
	50.00	0.99	99.68	0.93	99.87	98.88	0.53
Myricetin	4.16	0.67	100.63	0.55	99.35	98.78	0.71
	16.65	1.12	101.23	0.78	99.91	99.58	0.93
	133.2	0.89	99.78	0.95	100.01	99.51	0.58
Avicularin	0.08	1.02	98.75	1.06	100.63	100.15	0.47
	1.29	0.78	101.78	0.97	99.12	99.59	0.70
	5.19	0.94	100.1	0.71	99.95	99.41	0.93

**TABLE 4:** Specificity of 11 quantified compounds.

Phenolic acids			Tannins		
Chlorogenic acid: 11.95 min λ_max_: 218; 326 nm	Ellagic acid: 22.48 min λ_max_: 253; 354 nm	Gallic acid: 6.10 min λ_max_: 216; 271 nm	Oenothein A: 12.94 min λ_max_: 217; 264 nm	Oenothein B: 10.66 min λ_max_: 217; 364 nm	
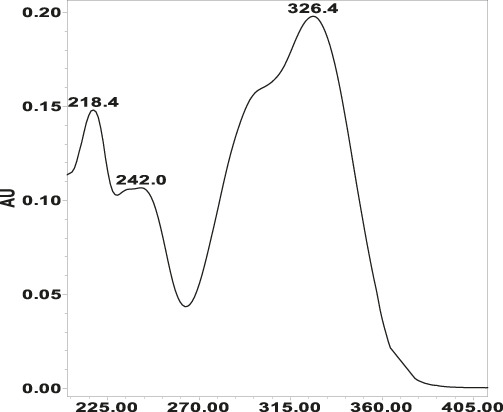	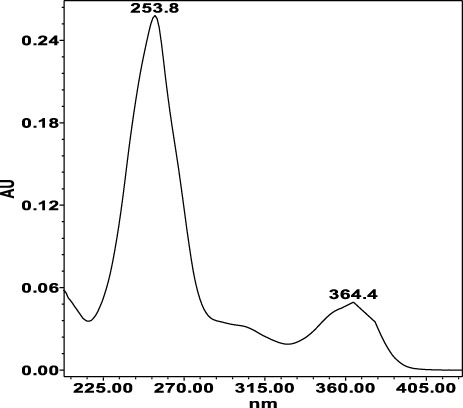	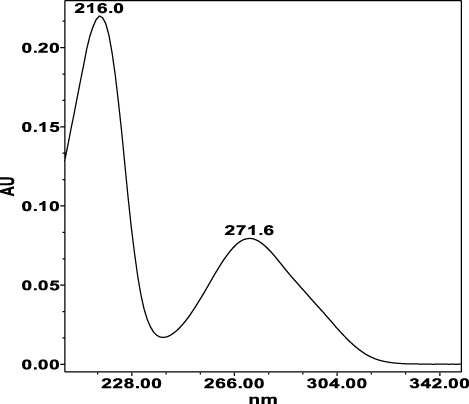	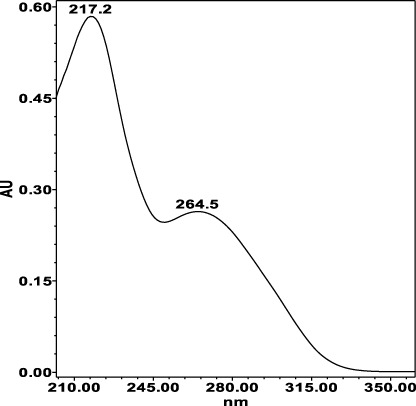	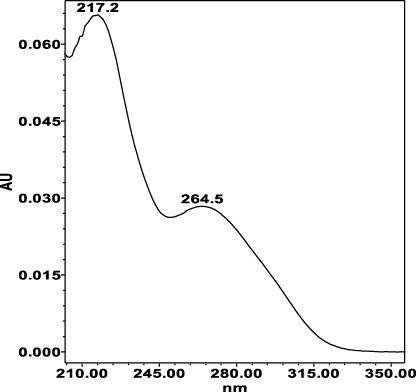	

Flavonoids					
Avicularin (=Quercetin 3-*α*-L-arabinofuranoside): 33.55 min λ_max_: 266, 327 nm	Guajaverin (=Quercetin 3-O-*α*-L arabinopyranoside): 28.31 min λ_max_: 256; 356 nm	Hyperoside (= Quercetin 3-O-*β*-D-galactopyranoside): 23.62 min λ_max_: 256; 353 nm	Isoquercitrin (=Quercetin 3-O-*β*-D-glucopyranoside): 24.50 min λ_max_: 256; 353 nm	Isomyricitrin (=Myricetin 3-O-*β*-D-glucopyranoside): 18.51 min λ_max_: 267; 357 nm	Myricetin (3 3′ 4′ 5 5′ 7-hexahydroxyflavone): 35.60 min λ_max_: 253; 376 nm
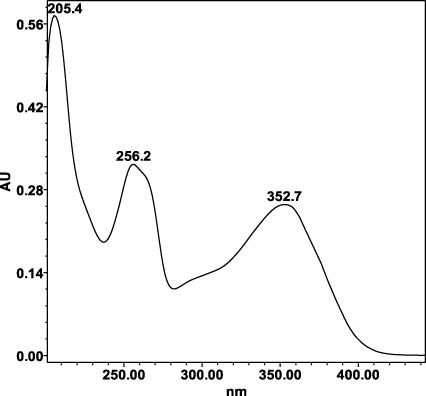	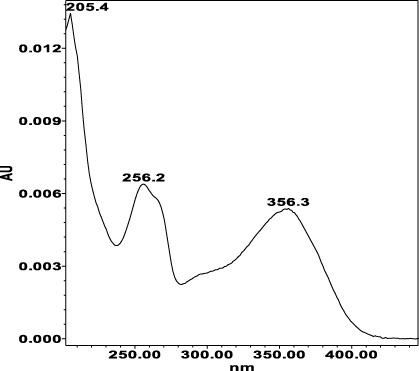	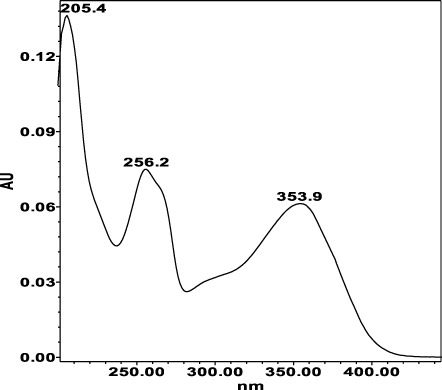	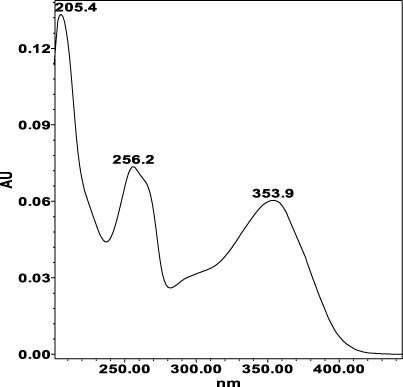	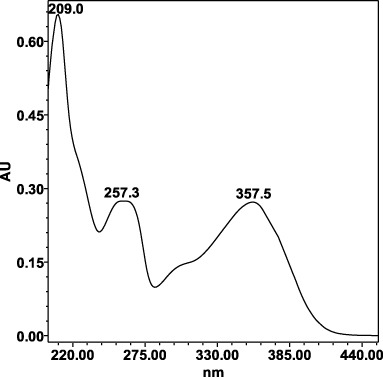	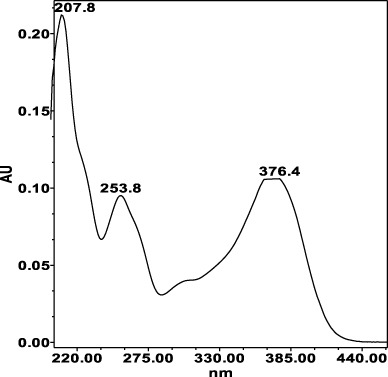

### 2.8 Statistical analyses

All data was processed using the LabSolutions Analysis Data System (Shimadzu Corporation). The results are mean ± standard deviation (SD) of three replicates (Microsoft Office Excel 2010, JAV). The value of *p* < 0.05 was taken as the significance level. The distribution of the data sets was assessed using the Shapiro-Wilk test. As all data were non-normally distributed, non-parametric statistical analysis methods were used. The significance level was set at *p* < 0.05. The Kruskal–Wallis test was used to detect differences between sets of samples, and the Dunn’s *post hoc* test was used for pairwise comparisons between samples. The effect of habitat, plant part and time of harvest of the raw material on the content of bioactive metabolites in the tissues was evaluated by two-way permutation analysis of variance (Euclidean similarity index and 9,999 permutations). Statistical analysis of the data was performed using PAST 5.0.2 software (Hammer, Harper, and Ryan, 2001).

## 3 Results

### 3.1 Optimisation of plant material extraction

The optimal conditions for the extraction of components and the chromatographic system for analysis were optimised. As there are no official monographs for *Epilobium* species in the national pharmacopoeias, we adapted the HPTLC method to assess the qualitative profile of the metabolites.

Initially, the method for extraction of the phenolic compounds in the methanolic extracts of *E. hirsutum* raw materials was identified using a modified pharmacopoeial Thin-Layer Chromatography (TLC) method, as specified in the European Pharmacopoeia (Ph. Eur.) 9.0 *Gingo leaf* monographs (Wichtl, 2002). Given the selected standards of phenolic compounds, three solvent systems were used (Table 5). The first method (ethyl acetate-formic acid-purified water (68:8:8 *v/v*)) showed the best separation of plant components from hydroxycinnamic acids and flavonoids’ groups, and the standards were also clearly visible on the plate. The detection of the zones was achieved using diphenylboric acid aminoethyl ether P and Macrogol as derivatising reagents. Chromatograms were observed under UV light at 254 nm and 366 nm wavelengths and white light before and after derivatisation.

**TABLE 5:** Parameters used in developing the HPTLC method.

Sample weight	HRM-extractant	Solvents	System
0,01; 0,2; **0,5** g	**1:10**; 1:20; 1:50	water; 70% ethanol; methanol; **methanol 50%**; methanol 50%	**EA: Formic ac: Water (68:8:8)** Dichloromethane: Water Formic acid: Acetic acid (100:25:11:10:10) Formic ac: Acetic acid: Water (134: 15: 36 : 15)
Extraction time	Extraction temperature	Injection volume	
10; **20**; 30; 60 min	25°C; **40**°C	3, 5, **7** µL	

Please note that the bold parameters are those were considered and selected as best method of analysis in this study.

To select the optimal solvent for sample extraction, we compared the results of chromatographic analysis using water, 70% ethanol, 50% methanol, and 100% methanol extracts. The colour intensity of the identified compound zones was similar across extracts, but in the water, 70% ethanol and 100% methanol extracts, zone separation was relatively poor, likely due to intense chlorophyll extraction, which interfered with separation. Additionally, extraction at 40°C yielded better metabolite release. Therefore, 50% methanol was selected as the optimal solvent for extracting phenolics from *Epilobium*.

### 3.2 HPTLC analysis

**FIGURE 3:**
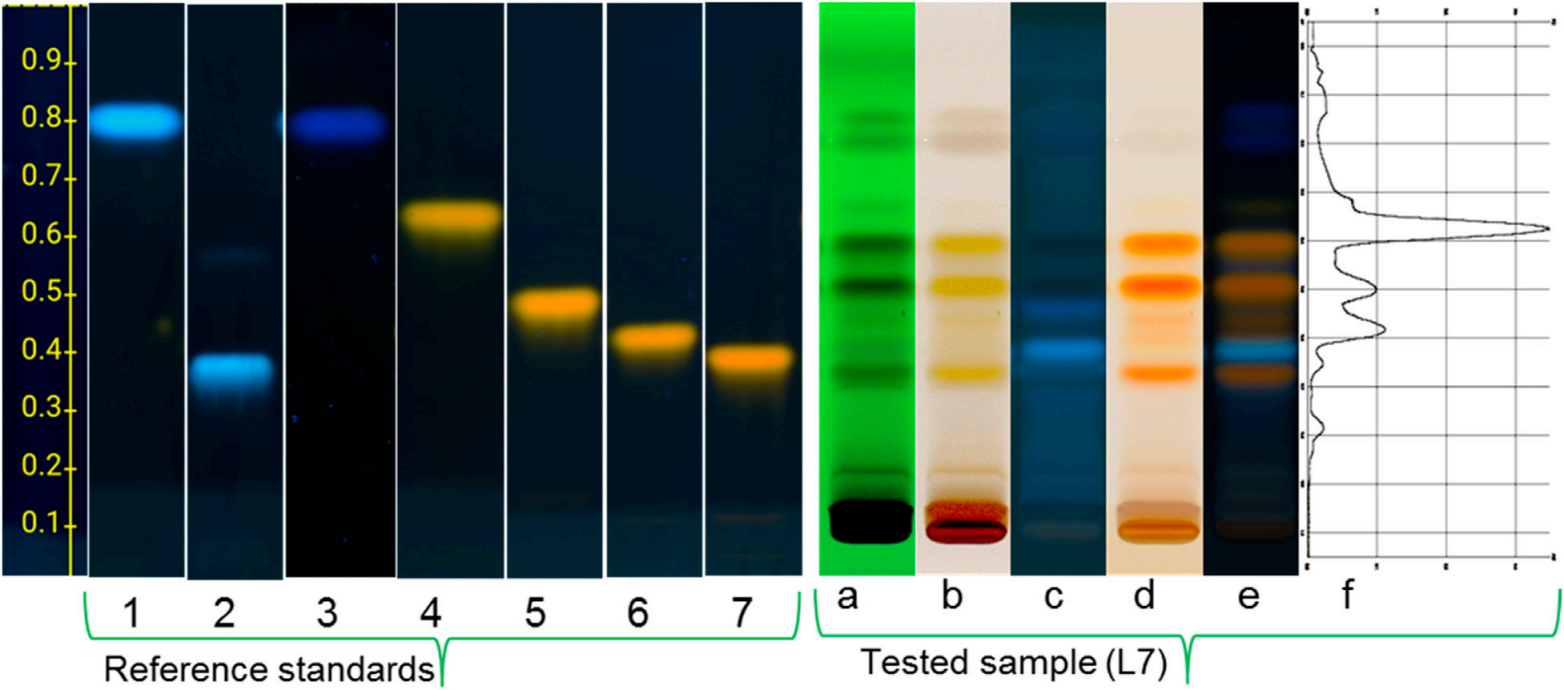
HPTLC of the reference standards zones (**1** – caffeic acid; **2** – chlorogenic acid; **3** – gallic acid; **4** – avicularin; **5** – guaijaverin; **6** – isoquercitrin; **7** – hyperoside) at wavelengths of 365 nm after derivatisation; and *Epilobium hirsutum* leaves extract (sample L7 from Grange Gardens, April 2024) at wavelengths of 254 nm **(a)**, in daylight **(b)** and 365 nm **(c)** before derivatisation and in daylight **(d)** and at 365 nm **(e)** after derivatisation and the total spectrum of zones at 365 nm **(f)**. System: EA: Formic acid: Water (68:8:8); derivatisation with diphenylboric acid aminoethyl ether followed by macrogol 400 and heating at 105°C for 5 min.

At UV 366 nm, the reference standards appear at the increasing retention factors (R_*f*_) values, e.g., hyperoside at R_*f*_ 0,41), isoquercitrin at (R_*f*_ 0,45), guaijaverin (R_*f*_ 0,52), avicularin (R_*f*_ 0,65) (Figure 3) with distinctive characteristic colours (yellow) respectively and after derivatisation at UV 366 nm with PEG and macrogol 400, those reference compounds appear yellow bands. The remaining reference standards, such as gallic acid (R_*f*_ 0,81), caffeic acid (R_*f*_ 0,87), (R_*f*_ 0,81) and chlorogenic acid (R_*f*_ 0,38), show good visibility under UV 254 nm and UV 366 nm.

**FIGURE 4:**
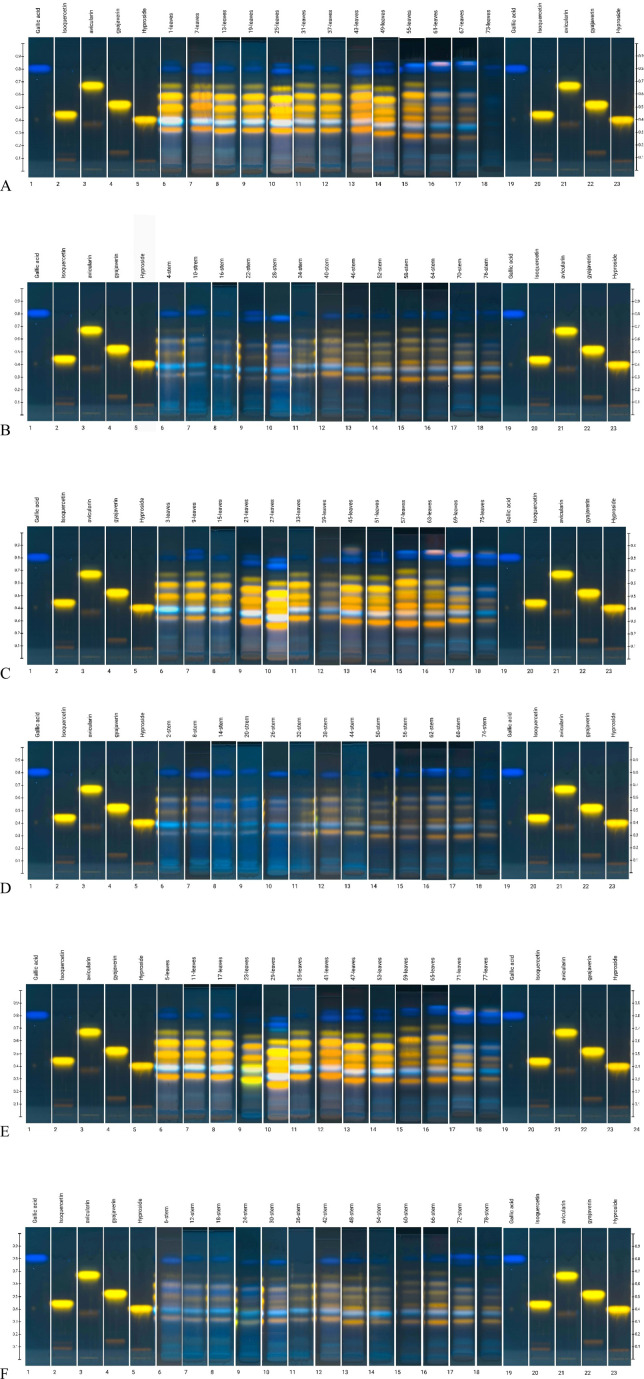
HPTLC profile of *Epilobium hirsutum* samples from **(A)** mesic grassland, leaves; **(B)** mesic grassland, stems; **(C)** lake shore, leaves; **(D)** lake shore, stems; **(E)** wet grassland, leaves; **(F)** wet grassland, stems after derivatisation at UV 366 nm. System: EA: Formic acid: water (68:8:8); derivatisation by diphenylboric acid aminoethyl ester followed by macrogol 400 and heating at 105°C 5 min.

HPTLC analysis showed a difference in chemical content between the leaves and stems of the plant (Figure 4). The methanol solution of the stems was light yellow, whereas the leaf extract was deep green. The chromatographic fingerprint showed very low levels of detectable constituents in the stems and the absence of caffeic acid, avicularin, guajaverin, isoquercitrin, hyperoside. The leaves contained all the components (caffeic acid, gallic acid, chlorogenic acid, avicularin, guajaverin, isoquercitrin, hyperoside) with visible differences in band intensity and colouration depending on location and the phenological phase. For example, hyperoside and isoquercitrin appeared as bright-yellow fluorescent zones under UV 366 nm, with increased intensity during the flowering stage in mesic grassland samples. Gallic acid was more prominent in early vegetative stages, typically appearing as a bluish-green zone with moderate intensity, especially in wet grassland specimens.

**FIGURE 5:**
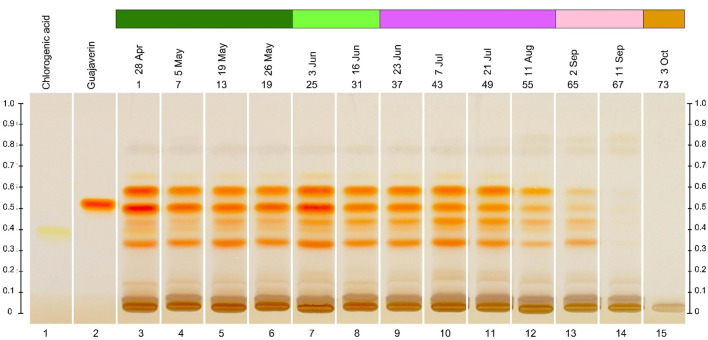
Dynamics of intensity of zones of compounds in different months of harvesting *Epilobium hirsutum* leaves with flower and leafing phenology data. The colour chart indicating the phenological phases is explained in Figure 1.

It should be noted that based on the visual (i.e., qualitative) assessment of the intensity of zones of marker compounds at different stages of plant vegetation, the dynamics of accumulation, decline, and maximum component content were noted (Figure 5), which was subject to further HPLC analysis. This is especially visible on the chromatogram with white light after derivatisation.

Some challenges were encountered in accurately identifying oenothein B, which has an R_*f*_ of approximately 0.1 – a low value for exact identification. This low R_*f*_ is probably due to the large molecular size of this ellagitannin, which perhaps affects its mobility. It should also be noted that the available literature describing the analysis of *Epilobium* species by HPTLC does not provide visual TLC data for oenothein B. In this study, chromatograms showing the observed zone separations are published for the first time.

### 3.3 Quantity and dynamics of metabolites by HPLC analysis

The dynamics of the accumulation of phenolic compounds in *E. hirsutum* samples was analysed at different phenological stages to determine the maximum period of their accumulation. Samples were collected from three different habitats and the environment definitely plays a key role in the accumulation of metabolites.

**FIGURE 6:**
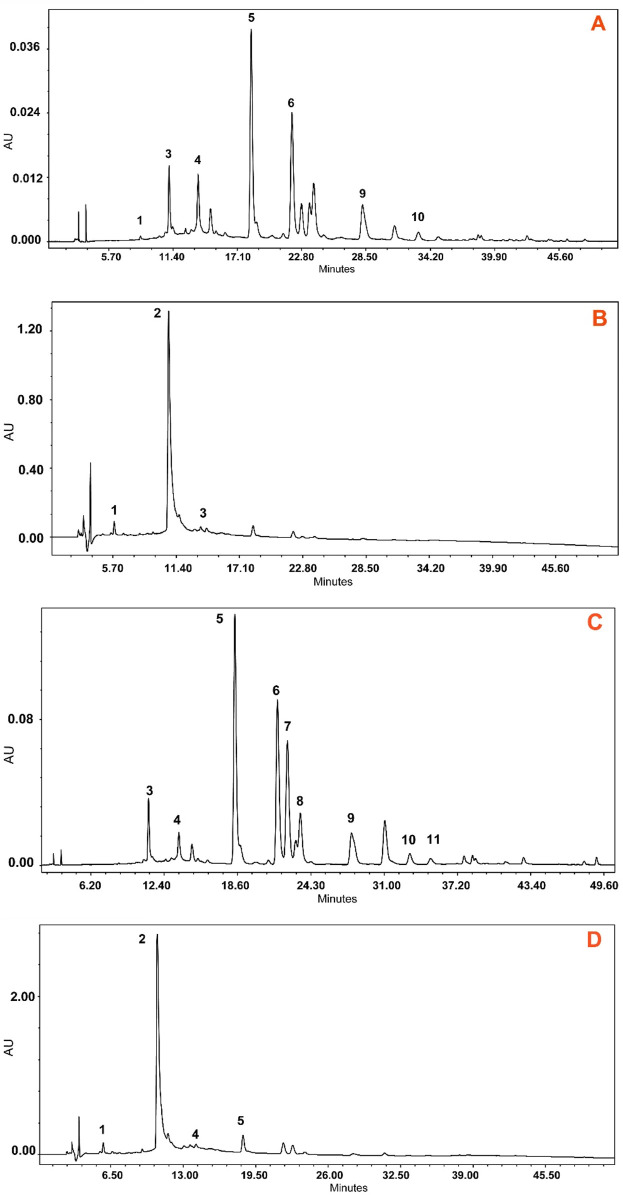
HPLC-DAD chromatograms of methanol (50%) extracts of *Epilobium hirsutum* stems (sample 44S–**A**, **B**) and leaves (sample 43L–**C**, **D**) from a mesic grassland habitat collected on 7 July 2023. The chromatograms were recorded at 350 nm **(A and C)** for identified polyphenols and at 219 nm **(B and D)** for tannins. Peaks: 1 – gallic acid; 2 – oenothein B; 3 – chlorogenic acid; 4 – oenothein A; 5 – isomyricitrin; 6 – ellagic acid; 7 – hyperoside; 8 – isoquercitrin; 9 – guajaverin; 10 – avicularin; 11 – myricetin. In chromatogram C, compound 8 partially coeluted with a minor peak. Identification was confirmed by retention time and UV-Vis spectrum matching the standard. Manual peak integration was used for accurate quantification.

The applied HPLC method showed a higher separation of compounds with good peak symmetry. In our study, we used 50% methanol as the extraction solvent which provides a higher extraction yield of highly polar compounds such as phenols and glycosides and an ultrasonic bath to enhance the extraction efficacy (Uminska et al., 2025). Chromatographic separation of the extracts was carried out using a Waters e2695 Alliance HPLC system equipped with an ACE C_18_ column. Gradient elution was applied with 0.1% trifluoroacetic acid in pure water and acetonitrile with increasing polarity from 5% to 95%. The HPLC fingerprint of the compounds is shown in Figure 6.

The developed method was fully validated according to the ICH Q2 (R1) guidelines. All standards and templates were introduced in three integrations. High reproducibility and low standard error were observed in multiple injections. All compounds showed good linearity (*r* ^2^ ≥ 0.997) across the tested ranges (Table 2). The method demonstrated satisfactory repeatability and precision, with RSD values for retention time and peak area ranging from 0.1% to 1.2%. The limits of detection (LOD) and limit of quantification (LOQ) were determined based on signal-to-noise ratios of 3:1 and 10:1, respectively.

In addition, the precision and accuracy at the LOQ level were assessed to confirm the method’s suitability for quantifying low-concentration analytes. The accuracy of the method was confirmed by a regeneration experiment. A sample of *Epilobium* extract impregnated with a known amount of a standard compound was analysed. The intra- and inter-day variation of the analysis was found to be less than 0.24%, and the maximum RSD did not exceed 1.31%, and recovery values ranged from 97.8% to 101.91%, with RSDs below 1.05%, demonstrating high trueness, repeatability, and robustness of the analytical procedure. The analysis was carried out in triplicate on three different days and each concentration point was given in triplicate (Table 3).

The identification of analytes in *Epilobium* samples was done by comparing the peaks retention times and the UV-spectrum obtained from the chromatogram of the standard solution (Table 4). Results revealed repeatability, accuracy, high sensitivity and good linearity of the method.

#### 3.3.1 Phenolic acids

Three phenolic acids were identified in the leaves and stems of *E*. *hirsutum*: chlorogenic acid, ellagic acid and gallic acid. Evaluation of the effects of habitat type, plant part, and time of sampling on phenolic acid content showed that chlorogenic and ellagic acid contents were significantly affected by factors different from gallic acid content.

The results of the two-way permutation analysis showed that the plant part had a significant effect on chlorogenic acid (F = 30.46, p = 0.0001) and ellagic acid (F = 11.87, p = 0.0007) but no significant effect on gallic acid (F = 1.26, p = 0.2720). Habitat type had no effect on the content of chlorogenic acid (F = 0.58, p = 0.5839) and ellagic acid (F = 1.40, p = 0.2600) but had a significant effect on the content of gallic acid (F = 3.64, p = 0.0348). The sampling date of *E*. *hirsutum* had no significant effect on the content of chlorogenic acid (F = 1.58, p = 0.1246) but had a significant effect on the content of ellagic acid (F = 2.23, p = 0.0249) and gallic acid (F = 2.05, p = 0.0375). The content of phenolic acids was not significantly affected by the interaction between habitat and plant part, habitat and sampling date, and sampling date and plant part.

**FIGURE 7:**
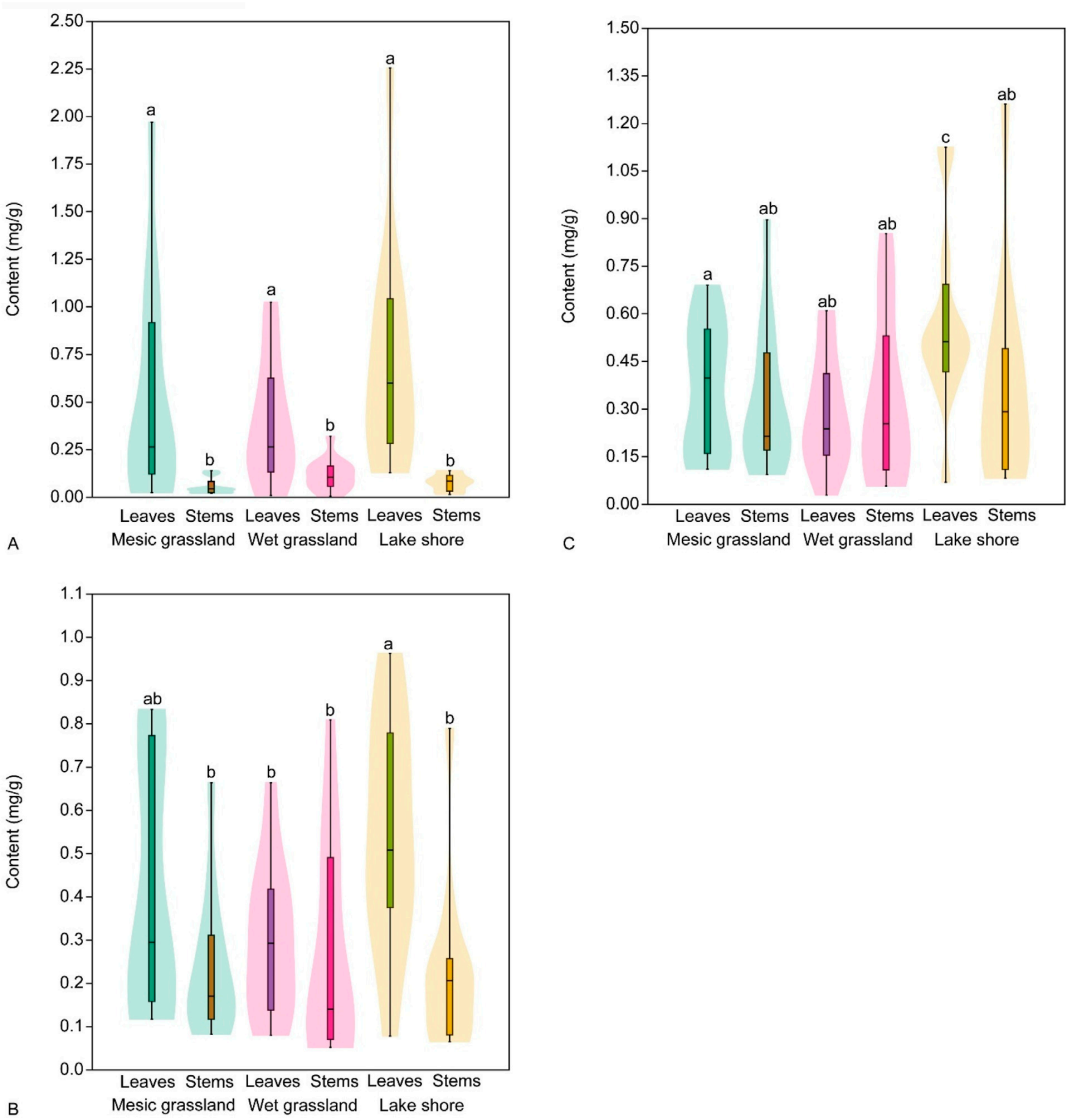
Mean content (mg/g) of phenolic acids, chlorogenic acid **(A)**, ellagic acid **(B)** and gallic acid **(C)**, in leaves and stems of *Epilobium hirsutum* in different habitats. Different letters above the violines indicate significant difference between samples according to the pairwise comparisons applying the Dunn’s *post hoc* test (p < 0.05).

The analysis of phenolic acid content in stems and leaves of *E*. *hirsutum showed* similar patterns throughout the study period (regardless of the date of sample collection). The content of chlorogenic and ellagic acids was significantly higher in leaves than in stems, except for ellagic acid, the content of which was not significantly different between leaves and stems in the wet grassland habitat (Figure 7; Table 6). No significant differences were found in the gallic acid content of leaves and stems, except in the leaves of plants growing in the lake shore habitat. These had a significantly higher gallic acid content (0.57 ± 0.29 mg/g DW) than the leaves and stems of plants growing in the other two habitats.

**TABLE 6:** Content (mean ± SD, mg/g) of biologically active metabolites analysed in stems and leaves of *Epilobium hirsutum* according to habitat type.

Compound	Mesic grassland		Wet grassland		Lake shore	
	Leaves	Stem	Leaves	Stem	Leaves	Stem
Phenolic acids						
Chlorogenic acid	0.51 ± 0.57^a^	0.06 ± 0.04^b^	0.39 ± 0.32^a^	0.12 ± 0.08^b^	0.70 ± 0.57^a^	0.08 ± 0.04^b^
Ellagic acid	0.41 ± 0.30^ab^	0.24 ± 0.16^b^	0.30 ± 0.17^b^	0.26 ± 0.25^b^	0.55 ± 0.26^a^	0.22 ± 0.19^b^
Gallic acid	0.36 ± 0.21^a^	0.32 ± 0.23^ab^	0.29 ± 0.18^ab^	0.33 ± 0.26^ab^	0.57 ± 0.29^c^	0.37 ± 0.32^ab^
Flavonoids						
Avicularin	0.14 ± 0.11^a^	0.00 ± 0.01^b^	0.14 ± 0.08^a^	0.02 ± 0.04^b^	0.19 ± 0.09^a^	0.01 ± 0.02^b^
Guajaverin	0.26 ± 0.24^a^	0.05 ± 0.04^b^	0.33 ± 0.25^a^	0.05 ± 0.08^b^	0.59 ± 0.45^a^	0.09 ± 0.09^b^
Hyperoside	0.46 ± 0.39^a^	0	0.59 ± 0.54^a^	0	0.91 ± 0.79^a^	0
Isomyrcitrin	0.56 ± 0.45^a^	0.09 ± 0.06^b^	0.76 ± 0.56^a^	0.19 ± 0.25^b^	0.96 ± 0.66^a^	0.14 ± 0.09^b^
Isoquercitrin	0.69 ± 0.89^a^	0	1.41 ± 1.96^a^	0	1.21 ± 1.39^a^	0
Myricetin	0.02 ± 0.02^a^	0	0.01 ± 0.04^a^	0	0.09 ± 0.09^b^	0
**Tannins**						
Oenothein A	0.12 ± 0.10^a^	0.06 ± 0.05^b^	0.70 ± 2.05^d^	0.75 ± 2.31^d^	0.25 ± 0.32^c^	0.06 ± 0.04^b^
Oenothein B	31.44 ± 24.75^a^	9.69 ± 5.01^b^	31.54 ± 23.34^a^	21.47 ± 14.98^c^	41.23 ± 21.33^a^	10.73 ± 5.24^b^

Different superscript letters in the row indicate significant differences between samples according to the results of Dunn’s *post hoc* pairwise comparisons.

**FIGURE 8:**
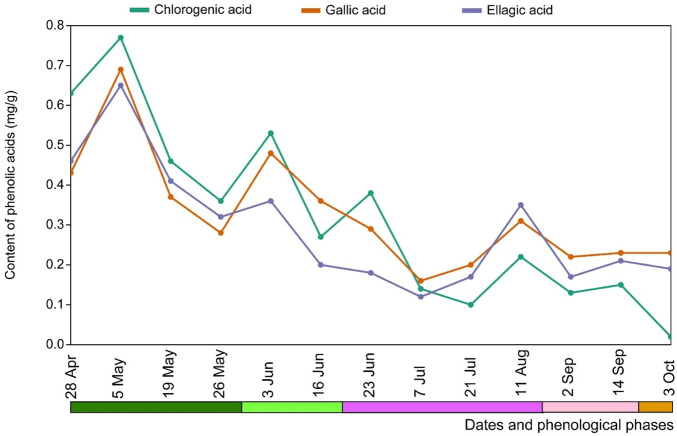
Mean content (mg/g) of phenolic acids in leaves of *Epilobium hirsutum* at different sampling dates and phenological phases in all habitats combined. The colour chart indicating the phenological phases is explained in Figure 1.

The analysis of the phenolic acid content in the leaves of *E*. *hirsutum* throughout the study season, depending on the date of collection but independent of the habitat type, showed that the highest and significantly higher contents were found at the end of the intensive growth period, with a fluctuating and gradual decrease thereafter, but with a significantly lower phenolic acid content compared to the intensive growth period only (Figure 8).

Comparative analysis of the phenolic acids content in *Epilobium* organs showed that in the stems the content of individual acids did not exceed 0.8 mg/g DW. Except for the gallic acid content in the 10S-sample from the lake shore (May 10) with a value of 1.26 mg/g DW. At the same time, the content of phenolic acids in the leaves reached 1.07–2.25 mg/g for chlorogenic acid in the 3L- and 9L-samples from lake shore (5 May – 28 April); in 11L-sample from wet grassland was 1.03 mg/g DW (May 05) and in 1L- and 7L-samples from mesic grassland 1.13–1.97 mg/g DW (28 April – 5 May) respectively. Caffeic acid was not determined by the HPLC method, probably due to its low amount or presence in the form of metabolite derivatives. The dominant acid in the *E. hirsutum* leaves was chlorogenic acid, while gallic acid and ellagic acid were the predominant acids in stem samples.

#### 3.3.2 Flavonoids

Six flavonoids were identified in the leaves and stems of *E. hirsutum*: avicularin, guajaverin, hyperoside, isomyricitrin, isoquercitrin and myricetin. Evaluation of the effects of habitat type, plant part and time of sampling on flavonoid content showed that all were similarly affected by the factors analysed, except for guajaverin and myricetin content.

The results of the two-way permutation analysis showed that plant part had a significant effect on the content of avicularin (F = 81.10, p = 0.0001), hyperoside (F = 46.95, p = 0.0001), isomyricitrin (F = 42.33, p = 0.0001) and isoquercitrin (F = 21.65, p = 0.0001) but they were not affected by habitat type (p > 0.05) and the interaction of plant part and habitat type (p > 0.05). Guajaverin content was significantly affected by plant part (F = 38.40, p = 0.0001) and habitat (F = 4.23, p = 0.0137), but not by the interaction of these factors (F = 2.56, p = 0.0803). Myricetin content was significantly affected by plant part (F = 32.85, p = 0.0001), habitat type (F = 5.68, p = 0.0024) and their interaction (F = 5.69, p = 0.0019).

Sampling date of *E. hirsutum* had no significant effect on the content of guajaverin (F = 1.23, p = 0.2375), hyperoside (F = 1.51, p = 0.1293), isomyricitrin (F = 1.90, p = 0.1678) and myricetin (F = 1.89, p = 0.1678). The content of avicularin (F = 1.77, p = 0.0742) was not significantly affected by sampling date, but the interaction of plant part and sampling date had a significant effect on its content (F = 2.82, p = 0.0055). Isoquercitrin content was significantly affected by plant part (F = 31.36, p = 0.0001), sampling date (F = 2.37, p = 0.0178) and their interaction (F = 2.38, p = 0.0168). Analysis of the effect of habitat type and sampling date showed that these factors and their interactions had no significant effect on the content of any flavonoids analysed.

**FIGURE 9:**
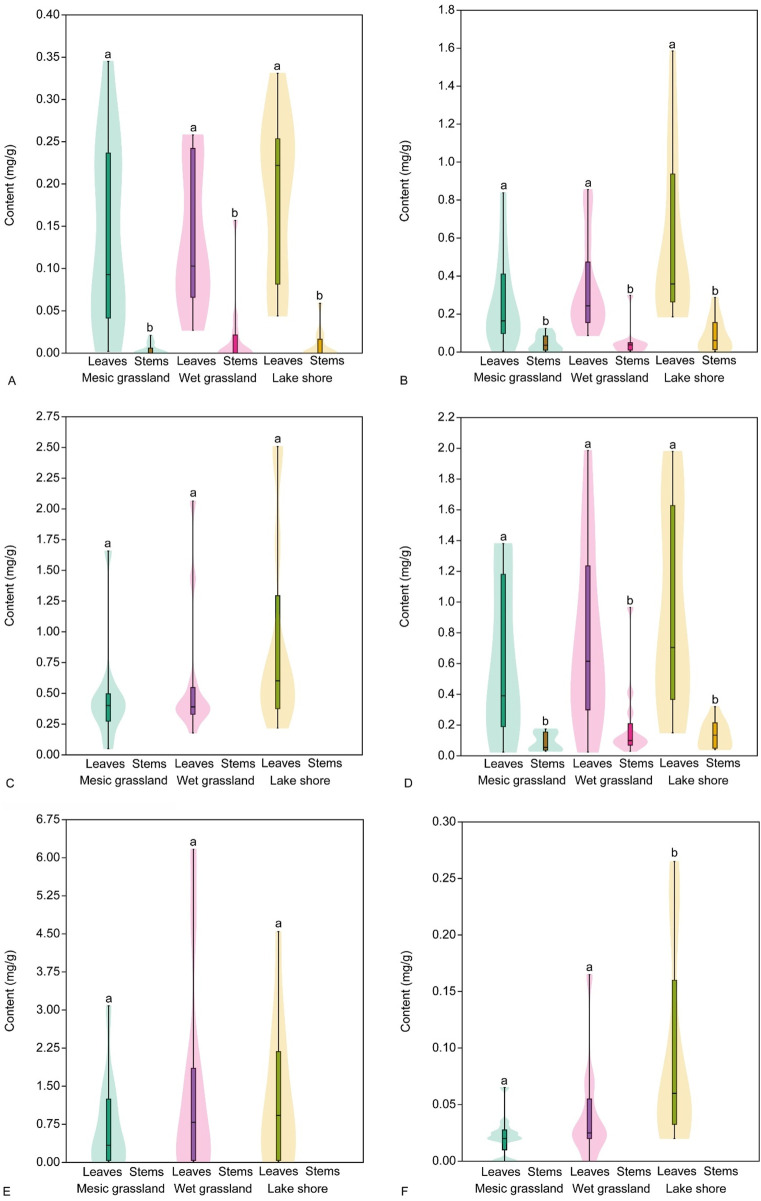
Mean content (mg/g) of flavonoids, avicularin **(A)**, guajaverin **(B)**, hyperoside **(C)**, isomyricitrin **(D)**, isoquercitrin **(E)** and guajaverin **(F)**, in leaves and stems of *Epilobium hirsutum* in different habitats. Different letters above the violines indicate significant difference between samples according to the pairwise comparisons applying the Dunn’s *post hoc* test (p < 0.05).

The analysis of flavonoid content in stems and leaves of *E*. *hirsutum* showed similar patterns to the phenolic acid content. Three flavonoids (hyperoside, isoquercitrin and myricetin) identified in leaves were absent in stems or their levels were below the threshold required for detection by the methods used in this study (Figure 9). No significant differences were found between the content of hyperoside and isoquercitrin in the leaves of *E. hirsutum*, regardless of habitat type, but the content of myricetin was significantly higher in the leaves of plants growing in the lakeshore habitat (Table 6). The content of avicularin, guajaverin, and isomyricitrin was significantly higher than that of the stems, and there were no significant differences between plants from different habitats. The mean content of all flavonoids, except isoquercitrin, was higher in leaves of plants growing in the lake shore habitat, although the differences from other habitats were not significant.

**FIGURE 10:**
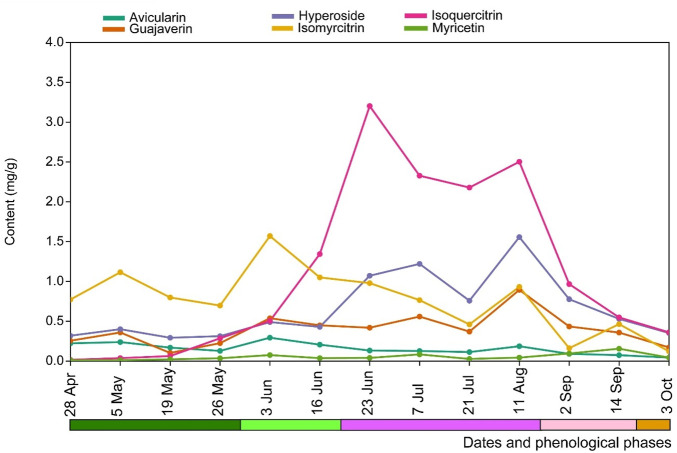
Mean content (mg/g) of flavonoids in leaves of *Epilobium hirsutum* at different sampling dates and phenological phases in all habitats combined. The colour chart indicating the phenological phases is explained in Figure 1.

The analysis of the flavonoid content in the leaves of *E. hirsutum* over the whole study period, depending on the date of collection but independent of the habitat type, showed that the content of avicularin and myricetin fluctuated little over the whole study period from spring to autumn. The content of guajaverin increased significantly from the beginning of the intensive growth period until flowering, and later its content changed insignificantly until the end of the wilting period (Figure 10). The levels of hyperoside and isomyricitrin were fluctuating, but their significant increase was noted at the end of flowering and flower formation periods, respectively. The most significant increase in the content of isoquercitrin was observed at the beginning of flowering and it remained at a high level until the end of flowering and the beginning of seed dispersal (Figure 10). The total content of flavonoids in the leaves of *E*. *hirsutum* was highest throughout the flowering season and was significantly higher than at other plant development stages.

The separation and identification of flavonoids using the sample *E. hirsutum* stems (A, B) and leaves (C, D) from a mesic grassland habitat collected on 7 July 2023 as an example is shown in Figure 6. In Figure 6C, the peak corresponding to isoquercitrin (8) exhibited partial coelution with a minor unidentified compound, resulting in a shoulder on the main peak. Despite the incomplete baseline separation, the identity of isoquercitrin was confirmed by comparing its UV-Vis spectra obtained with the DAD detector to the authentic reference standard. Quantification was performed based on the spectral purity and peak apex matching, as no spectral interference was observed within the characteristic absorbance range of isoquercitrin (λ_max_ ≈ 254 and 354 nm).

#### 3.3.3 Tannins

Two tannins, oenothein A and oenothein B, were identified in the leaves and stems of *E*. *hirsutum*. Their concentrations varied according to habitat type, plant organ and time of collection. Due to their polyphenolic structure, both compounds are moderately soluble in polar solvents (e.g., water, ethanol), but may have limited stability under prolonged heating or high pH conditions, which should be considered during extraction and formulation.

The results of the two-way permutation analysis showed that plant part had no significant effect on the content of oenothein A (F = 0.06, p = 0.5420), but it had a significant effect on the content of oenothein B (F = 26.72, p = 0.0001), whereas habitat type and its interaction with plant part had no significant effect on the tannin content. The same regularity was found when analysing the effect of plant part and sampling date. Both factors had no significant effect on the content of oenothein A, but the content of oenothein B was significantly influenced by plant part (F = 30.67, p = 0.0002). The effect of both factorial interactions had no significant effect on the content of tannins. However, when analysing the effect of habitat and sampling date, a significant effect of habitat type (F = 83.12, p = 0.0084), sampling date (F = 80.01, p = 0.0122) and their interaction (F = 84.92, p = 0.0111) was found on the content of oenothein A, but these factors and their interactions had no significant effect on the content of oenothein B.

**FIGURE 11:**
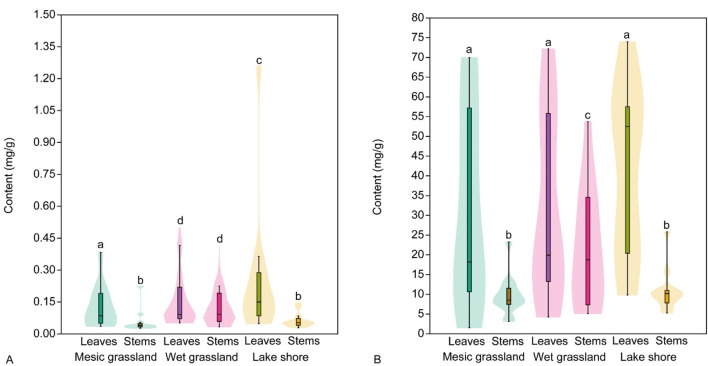
Mean content (mg/g) of tannins, oenothein A **(A)** and oenothein B **(B)**, in leaves and stems of *Epilobium hirsutum* in different habitats. Different letters above the violines indicate significant difference between samples according to the pairwise comparisons applying the Dunn’s *post hoc* test (p < 0.05).

The analysis of tannin content in stems and leaves of *E. hirsutum* showed somewhat different patterns to the accumulation of other metabolites. The content of oenothein A differed significantly between habitats, with the highest content found in leaves and stems of wet grassland plants (0.70 ± 2.06 mg/g and 0.75 ± 2.31 mg/g DW, respectively). In contrast, in other habitats its content was significantly lower in both leaves and stems (Table 6). The lowest levels of oenothein A were found in leaves and stems of plants from mesic grassland (Table 6; Figure 11). The content of oenothein B in leaves and stems followed the same pattern as that of phenolic acids and flavonoids. The content of oenothein B was significantly higher in leaves than in stems and no significant differences were found between habitats. However, the content of oenothein B was significantly higher in stems of plants from wet grassland than in stems from mesic grassland and lake shore habitats (Table 6).

**FIGURE 12:**
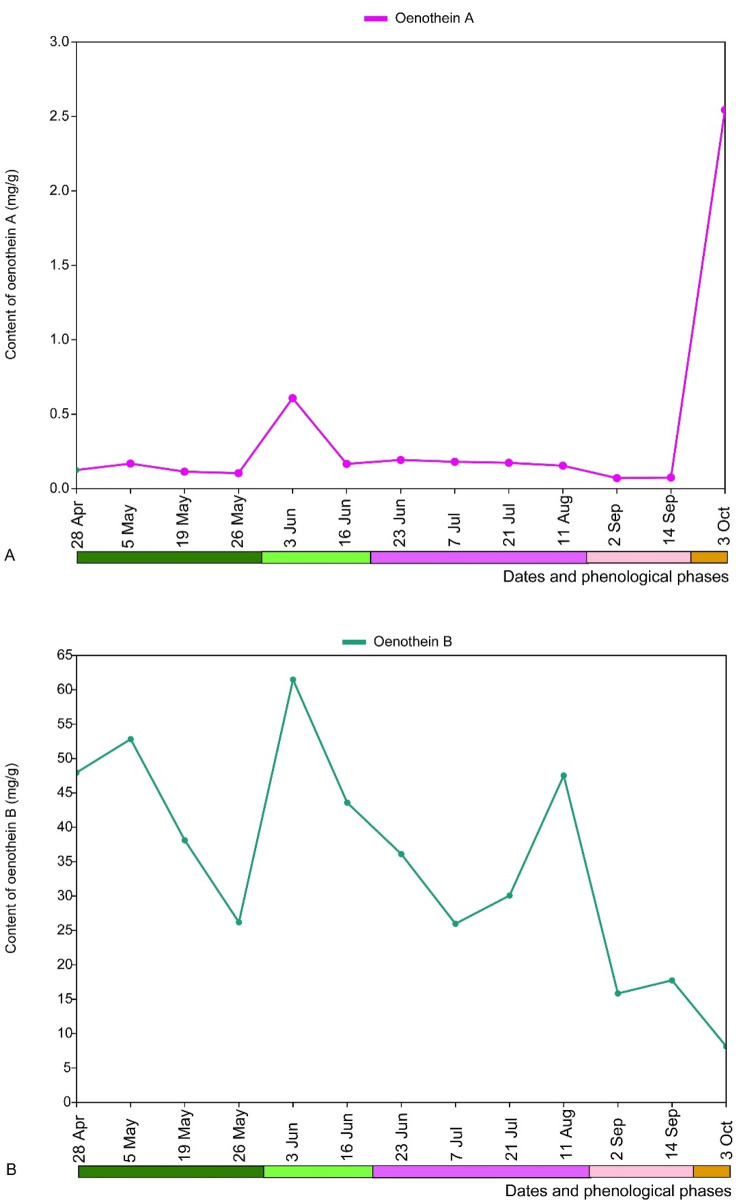
Mean content (mg/g) of tannins, oenothein A **(A)** and oenothein B **(B)**, in leaves of *Epilobium hirsutum* at different sampling dates and phenological phases in all habitats combined. The colour chart indicating the phenological phases is explained in Figure 1.

The analysis of the tannin content in the leaves of *E*. *hirsutum* during the whole study period, depending on the time of collection but independent of the habitat type, showed that the highest and significantly higher content of oenothein A was found in the wilting stage of the plant, while its content fluctuated insignificantly during the whole study period (Figure 12). The opposite tendencies were observed for the changes in oenothein B content: during the entire growth and flowering period its content was high, although fluctuating insignificantly, but at the time of seed dispersal and wilting its content decreased significantly compared to the previous phenological phases (Figure 12).

**FIGURE 13:**
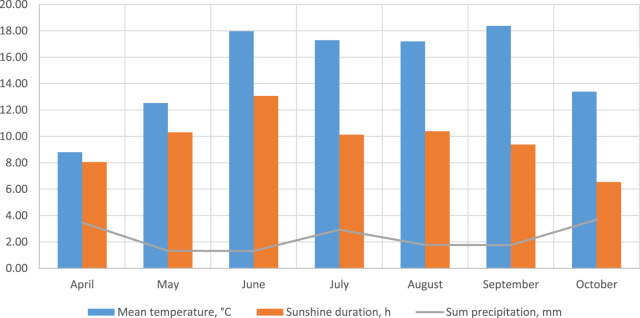
Monthly overview for 2023 of mean temperature, sunshine duration (average per day and the total precipitation per months) at Wallington area, United Kingdom. A more detailed breakdown can be found in Supplementary Table S1.

Meteorological data recorded during the study period (April–October 2023) showed patterned variations in temperature, precipitation, and sunshine duration (see Supplementary Table S1; Figure 13). Higher precipitation in early Spring coincided with increased accumulation of phenolic acids, while prolonged dry periods in summer were correlated with increased levels of flavonoids and tannins, likely due to stress adaptation mechanisms.

## 4 Discussion

A comparative analysis of the phenological phases of *E. hirsutum* and the ontogeny of plants allows us to identify the characteristics of accumulation marker compounds and, thus, identify the optimal phases and plant organs for appropriate collection times of plant raw materials for pharmaceutical applications.

The phenological development of *E. hirsutum* is strongly related to the biosynthesis of metabolites and is consistent with known metabolic pathways. Hydroxycinnamic acids, abundant during the growth phase, decrease as the plant progresses towards flowering, where flavonoids become more abundant (Khawula et al. 2023). This transformation will likely support increased biological activity and plant protection mechanisms during the reproductive phase. In addition, the HPTLC method complemented the HPLC data to confirm the presence of key marker compounds and quantify their concentrations depending on the vegetation stage, habitats, collection periods and plant part.

Traditionally, *Epilobium* is harvested by cutting 2/3 of the top of the plant, including stems and leaves, crushing it and drinking it as a tea. However, our research shows that the quantity of relevant metabolites in the leaves significantly exceeds that in the stems. The observed variations in compound content across organs and stages support the importance of identifying optimal collection plots for maximising phytochemical yield to produce the same quantity of relevant metabolites and/or marker compounds. Optimised cultivation and harvesting methods can lead to more stable and higher yields, providing a steady supply of plant raw materials for the pharmaceutical industry.

### 4.1 Phenolic acids

During the early stages of plant development, chlorophyll and secondary metabolites are produced due to photosynthetic activity. Therefore, this stage is expected to have high levels of phenolic compounds, mainly phenolic acid derivatives (Kumar and Goel, 2019), typically synthesised in young plant tissues. These metabolites are primarily derived from the shikimic acid and phenylpropanoid pathways. However, at this stage, the biomass is still too low for large-scale collection for the pharmaceutical industry.

During the intensive growth phase*, E. hirsutum* focuses on vegetative growth, producing leaves and stems. During this phase, the accumulation of marker compounds (particularly chlorogenic acid and gallic acid) is generally associated with the plant’s defence mechanisms against oxidative stress and growth regulation. The primary phenolic acids in the tissues of *E. hirsutum* plants, gallic, ellagic and chlorogenic acids, influence the formation of tannins in the plant’s raw material (Jürgenson, Matto, and Raal, 2012). In general, the accumulation pattern during the vegetation phases in the three different habitats was the same; namely, the highest concentration was observed during the growth phase with a gradual decrease in content towards the seed formation phase. The highest accumulation was found during the growth phase (April-May), and a decrease was observed during the flowering phase (June-July-August), probably due to their conversion into flavonoids and lignin precursors. During the seed formation phase (September-October), the content of phenolic acids did not exceed 0.2 mg/g DW for stems and 0.8 mg/g DW for leaves. Compared to well-lit dry habitats, higher concentrations were observed in plants collected from shaded wet grasslands and lakeshores.

Hydroxycinnamic acid content varied between habitats and growth stages, highlighting the adaptability of *E. hirsutum* to environmental conditions. Hydroxycinnamic acid content varied across habitats and growth stages, highlighting the adaptability of *E. hirsutum* to environmental conditions. For example, elevated levels of chlorogenic acid were noted in samples from shaded habitats. Changes in the accumulation of phenolic compounds in *E. hirsutum* were strongly influenced by meteorological conditions. In our analysis, increased precipitation in early spring (up to 3.47 mm in April, Supplementary Material, Figure 13) promoted the synthesis of phenolic acids in *E. hirsutum*, consistent with results obtained in *E. angustifolium* harvested from the Carpathian Mountains, Ukraine, 2019, which reported similar trends in hydroxycinnamic acid dynamics under varying phenological phases using the same HPLC method (Ivanauskas et al., 2024). In another study, samples of *E. angustifolium* herb samples collected during flowering (June 2015) and after flowering (September 2015) were analysed (Gryszczyńska et al., 2018). In the samples collected in September, the gallic acid content was 7.04 mg/100 g, while the June samples had a gallic acid content of 3.91 mg/100 g by UPLC-MS/MS. The plants were grown *in vitro* cultures and micro-propagated from seeds from Germany and Poland; the resulting roots were transferred to a substrate and later planted in the soil. Therefore, in this case, the data comparison is not entirely correct due to the difference in the species and the nature of the origin of the samples. This is only a comparison of the dynamics of the formation of individual substances in representatives of the genus *Epilobium*.

However, analytical data of *E. hirsutum* aerial parts collected during the flowering period at the end of June from natural habitats of Iran (Mohammadi Bazargani, Falahati-Anbaran, and Rohloff, 2021) showed that gallic acid had the highest concentration among phenolic compounds (quinic acid, gallic acid, (E)-caffeic acid) and also among all other secondary metabolites with a mean value of 15.12 and 23.36 mg/g DW by GC/MS method after derivatisation with MSTFA reagent.

The metabolic variability of *E. hirsutum* highlights the importance of selecting the optimal growth stage, habitat, and plant part to maximise the desired metabolite yield for pharmaceutical applications. The results suggest that using *E. hirsutum* leaves rather than stems may be more feasible. Selection of appropriate habitats and simulation of favourable conditions (e.g., shaded environments) may enhance phenolic acid production, providing a sustainable and scalable source for industrial use.

In *Epilobium* species, gallic and ellagic acids serve as precursors of hydrolysable tannins, and particularly a cyclic dimeric ellagitannin oenothein B, the dominant pharmacologically active metabolite of this genus (Ramstead et al., 2012; Okuyama et al., 2021). These compounds accumulate during reproductive and senescent stages (Gasperotti et al., 2013) and exhibit significant antioxidant, anti-inflammatory, and anticancer properties (Marino et al., 2022). Their pharmacological potential extends to the prevention and treatment of cardiovascular diseases, neurodegenerative disorders (e.g., Alzheimer’s, Parkinson’s), and metabolic diseases such as diabetes.

### 4.2 Flavonoids

As *E. hirsutum* progresses through the growth and reproductive stages, phenolic acids often serve as precursors for more complex secondary metabolites, including flavonoids (Cheynier et al., 2013). Upon entering the flowering phase, the plant produces vibrant pink-purple flowers, signalling increased flavonoid biosynthesis. This phase also triggers the production of lignins and tannins, which are essential in UV protection, pigmentation, and reproduction. Flavonoids, in particular, attract pollinators and protect them from pathogens, underlining their ecological and pharmacological importance.

The accumulation of specific flavonoids such as avicularin, guajaverin, isoquercitrin, hyperoside, myricetin, and isomyricitrin in *E. hirsutum* is influenced by the growth stages of the plant, a phenomenon observed in several *Epilobium* species (Ak et al., 2021). The concentration of flavonoids increases as the plant matures, with the flowering stage often showing higher levels of these metabolites than earlier vegetative stages. This shift is likely to be related to the developmental priorities of the plant, with energy allocation shifting from growth to reproduction, promoting higher synthesis of secondary metabolites such as flavonoids during flowering. Therefore, the HPLC method is recommended for accurate quantitative analysis due to its high precision in measuring individual metabolites in complex plant mixtures.

Glycosylated flavonoids such as hyperoside and isoquercitrin were absent from *E. hirsutum* stems, and avicularin appeared only during the intense growth phase (April-May) and seed dispersal phase (September) but its content did not exceed 0.02 mg/g DW in samples. Guajaverin content in stems is very low, averaging no more than 0.05–0.1 mg/g DW across samples, gradually increasing with the onset of flowering (June) and peaking at 0.2–0.3 mg/g DW during seed formation (September).

The distribution of metabolites in descending order in *Epilobium* leaf samples is isoquercitrin > isomyricitrin > hyperoside > guajaverin > avicularin > myricetin. The maximum accumulation of these flavonoid glycosides occurs during the plant’s flowering period (June-August). The maximum accumulation of isoquercitrin found during this period is 1.40–3.08 mg/g DW.

The distribution of the compounds in the stems was the same regardless of the habitat type. However, in the plant samples from the wet grassland and the lake shore, the content of guajaverin was higher in two times (0.28–0.30 mg/g DW for 64S and 66S samples) compared to the mesic grassland (0.12 mg/g DW, 68S) in August-September. Of course, the qualitative and quantitative profile of metabolites in the leaf samples was significantly higher than in the stems, which is also in agreement with the data of other authors (Remmel et al., 2012) on the assessment of the total polyphenols, tannins and flavonoids content by UV spectroscopy in the organs (roots, stems, leaves, and flowers) of *E. hirsutum* and similar species. This highlights the importance of selecting appropriate plant parts for raw material collection to optimise secondary metabolite yield.

High levels of glycosylated derivatives of quercetin were observed in the *E. hirsutum* leaf samples collected from the wet grassland and lakeshore habitats, probably due to the moderate environmental light conditions and moisture availability, which favoured the accumulation of metabolites. In our case, samples collected from late May to June-July-August were exposed to the sunshine duration for an average of 10.13–13.07 h/day (Supplementary Material). It should be noted that in the samples (39L-75L) from the lake shore, the second dominant metabolite after isoquercitrin (1.05–4.54 mg/g DW) was hyperoside (0.6–2.5 mg/g DW), possibly due to increased exposure to sunlight and reduced competition.

Myricetin (0.03–0.265 mg/g) and its glucoside (0.04–1.985 mg/g) were also found in the samples and were reported in *E. hirsutum* from Estonia by (Ain et al., 2024) and from Turkey by (Ak et al., 2021) and Iran by (Mohammadi Bazargani, Falahati-Anbaran, and Rohloff, 2021).

The variability in flavonoid content between *E. hirsutum* samples highlight the influence of environmental factors, plant part, and phenological stage on secondary metabolite biosynthesis. Higher flavonoid levels during the active flowering phase in samples collected in shaded or moderately illuminated humid habitats suggest a balance between light-induced synthesis and regulation of oxidative stress. These results highlight the role of plant adaptation to the environment in secondary metabolite production, with implications for plant-based pharmaceutical applications.

Understanding the optimal timing of isoquercitrin accumulation is crucial as this flavonoid glycoside exhibits antioxidant, anti-inflammatory, cardioprotective (Valentová et al., 2014) and antiviral (Agrawal, Blunden, and Jacob, 2024) effects, making it beneficial for various health conditions. Most of the identified flavonoids are active constituents of many medicinal plants used in traditional medicine for their neuroprotective, anti-inflammatory, antioxidant, antiproliferative and other pharmacological properties (Schepetkin et al., 2016).

### 4.3 Tannins

After the flowering phase, *E. hirsutum* enters the seed development phase. During this phase, the plant focuses on seed maturation (Sripathy and Groot, 2023). Marker compounds associated with this phase include fatty acids, particularly *γ*-linolenic acid (Mendes et al., 2013), and tannins (Santos et al., 2021) such as oenothein B. During the later stages of plant development, metabolites are degraded, and their levels decrease significantly (up to 3–9 mg/g DW).

Oenothein A and B, the major macrocyclic ellagitannins, are the main bioactive components of *E. hirsutum* (Yoshida, Yoshimura, and Amakura, 2018), and are known for their antioxidant, immunomodulatory, antiviral, tumour cell cytotoxicity properties (Ramstead et al., 2012). Considering the renewable resources of the plant and the high content of oenothein B depending on the stage of vegetation, the development of herbal preparations based on *Epilobium* species is promising.

The content of oenothein A in *Epilobium* stems did not exceed 0.03–0.2 mg/g DW and was the highest in the *Epilobium* samples from the wet grassland habitat. It should be noted that in the sample 78S, collected in October in the wet grassland during the wilting phase, the content of oenothein A reaches the maximum value of all samples, namely, 8.43 mg/g DW, which can be attributed to the increased occurrence of rainfall during this period (3,69 mm of precipitation in October). In the leaves, the content of oenothein A was slightly higher than in the stems and was at the level of other phenolic compounds, ranging from 0.03 to 1.26 mg/g DW, with the maximum accumulation in June: 0.38 mg/g DW for the 25 L sample from the mesic grassland, 1.26 mg/g DW for the 27L sample from the lakeshore and 2.26 mg/g DW for the 41L sample from the wet grassland. As for the 78S sample of *E. hirsutum* stems from the wet grassland, the oenothein content in the 77L sample leaves during the rainy season in October was comparatively high at four times (7.52 mg/g DW).

The concentration of oenothein B in *E. hirsutum* samples was significantly higher than that of other phenolic compounds. It ranges from 5.11 to 53.75 mg/g DW for stems and from 4.27 to 73.97 mg/g DW for leaves, depending on the habitats and the development phase. The highest accumulation of oenothein B was observed in *E. hirsutum* samples collected during the flowering period of the plant in June in the United Kingdom, which correlates with the data of the authors (Jürgenson, Matto, and Raal, 2012) who studied the total tannin content in *E. angustifolium* organs (roots, stems, flowers) during the whole growth period from May to October in Estonia and indicated that the highest tannin content was found in small growing plants in May. The highest average temperature (up to 18 °C, Supplementary Material) is also observed in June and September in Wallington area, United Kingdom in 2023. Due to geographical differences between countries, the flowering period of the plant falls in different months (Sell and Murrell, 2009). Based on the comparative analysis, the above-ground organs without stems, collected in the United Kingdom in July-August, are the best choice for obtaining *E. hirsutum* plant material with a consistently high phenolic compound content.

Leaf samples from the mesic grassland had lower levels of oenothein B, possibly due to reduced water availability. Plants from the wet grassland and lakeshore habitats had the highest levels of oenothein B, reflecting optimal growth conditions, with samples from the lake shore habitat having slightly higher levels, possibly due to the higher light intensity, which favours ellagitannin biosynthesis (Niemetz and Gross, 2005). Generally, a pattern of biosynthesis of phenolic compounds was observed in the samples, with a decrease in the levels of phenolic acids and an increase in the levels of flavonoids and tannins as products of their transformation.

The biosynthesis of phenolic acids, flavonoids and tannins in *E. hirsutum* is regulated by environmental factors such as light intensity, temperature and water availability. For example, UV exposure is a known signal for flavonoid accumulation, particularly quercetin derivatives, which act as UV protectors and antioxidants. Similarly, phenolic acid synthesis is often increased in response to abiotic stress such as drought or oxidative stress, acting as part of the plant’s defence mechanism. In our study, the increased flavonoid and tannin levels observed in shaded or moist habitats suggest that moderate stress and light exposure may positively influence secondary metabolite accumulation, possibly through regulation of the phenylpropanoid pathway. In Understanding these variations is crucial for optimising raw material collection to ensure sustainable harvesting and high-quality pharmaceutical products.

Due to climate change, the predictability of crop seasons has become more uncertain in recent decades. Changes in meteorological conditions are the main factor affecting harvesting time, productivity, and the quality of plant raw materials (Yeleliere, Antwi-Agyei, and Guodaar, 2023). From 1880 to 2012, the planet’s temperature increased by 1.54°C (Copernicus, 2025). Heat waves, which are increasing in frequency and duration, can cause premature ripening of crops and medicinal plants, leading to changes in the yield and quality of plant raw materials.

The meteorological data for the selected sampling sites is very similar, as they were collected in the same county as England (Figure 13). It can only be noted that Lake Shore (site 2 in the Supplementary Material) and wet grassland (site 1) had higher average temperatures (during June–September 17°C–18°C) and slightly longer sunshine duration (up to 13 h in June) than mesic grassland (site 0), which was in a shaded area. However, all three sites showed similar trends in sunshine duration (from 6.5 to 13 h, depending on the month), indicating that they are exposed to comparable regional weather conditions. Lake shore showed slightly greater temperature variations, probably due to the influence of the lake, which moderates temperatures but can also create sharper contrasts. Precipitation was similar at all three sites, but soil moisture retention was longer at wet grassland and lake shore. General meteorological observations were as follows: (1) lake shore was the wettest and most variable due to the influence of water; (2) wet grassland has the most consistent wet conditions with slightly higher temperatures; (3) mesic grassland was the driest and coolest of the three, with more consistent conditions but less precipitation. These differences highlight how local ecosystems respond to different climates, affecting plant growth, soil moisture, and biodiversity at each site. While *E. hirsutism* can be sustainably sourced due to its weediness, our finding supports and highlights the importance of an integrative approach to ecological assessment and sustainable harvesting practices, including the development of targeted strategies to reduce waste and optimise resource use. Identifying the optimal phenological stages for harvesting reduces unnecessary collection of plant material, thereby conserving biodiversity and reducing environmental impact. The observed variability in bioactive compound accumulation across phenological stages and habitats highlights the need for evidence-based harvesting strategies. Our results support the need for individual harvesting protocols for *E. hirsutum*, with a focus on harvesting leaves during the flowering stage under moist, shaded habitats to maximise the yield of bioactive components and, as a consequence, the pharmacological potential of plant application.

## 5 Conclusion

The optimisation of *E. hirsutum* collection based on growing season and habitat is essential for optimised pharmaceutical production. Environmental factors such as water availability, light and temperature significantly influence the biosynthesis of relevant metabolites and/or marker compounds. This study demonstrates that the accumulation of key phenolic compounds in *E. hirsutum* is significantly influenced by phenological phase, plant organ and habitat type. Leaves consistently contained higher concentrations of all pharmacologically active marker compounds then stems, with the flowering phase showing the highest levels of flavonoids such (e.g., isoquercitrin and hyperoside). Chlorogenic acid accumulation was highest during early vegetative stage, particularly in shaded, moist habitats, while oenothein B was most abundant during flowering and in leaf samples from wet grassland and lakeshore habitats. Notable, mesic grassland favoured the accumulation of stress-related phenolics, while wetter habitats promoted increase biomass with generally lower secondary metabolite levels. By identifying these key patterns in metabolite distribution across habitats and phenological phases, this study contributes to a better understanding of chemical variability in *E. hirsutum*, which is essential for consistent pharmaceutical quality.

This study highlights the environmental benefits of targeted harvesting practices for *E. hirsutum*. It also highlights the need of providing detailed phytochemical profiles and full biogeographical and botanical data on an extract investigated (Heinrich et al., 2022). We show what is needed to optimise resources to support environmental conservation and efficient pharmaceutical production, ensuring the availability of high-quality, sustainable plant materials.

## Data Availability

The original contributions presented in the study are included in the article/Supplementary Material, further inquiries can be directed to the corresponding authors.
